# Selective aldehyde reductions in neutral water catalysed by encapsulation in a supramolecular cage†

**DOI:** 10.1039/d1sc00896j

**Published:** 2021-03-12

**Authors:** Avishek Paul, Michael A. Shipman, Dolapo Y. Onabule, Stephen Sproules, Mark D. Symes

**Affiliations:** WestCHEM, School of Chemistry, University of Glasgow University Avenue Glasgow G12 8QQ UK mark.symes@glasgow.ac.uk stephen.sproules@glasgow.ac.uk

## Abstract

The enhancement of reactivity inside supramolecular coordination cages has many analogies to the mode of action of enzymes, and continues to inspire the design of new catalysts for a range of reactions. However, despite being a near-ubiquitous class of reactions in organic chemistry, enhancement of the reduction of carbonyls to their corresponding alcohols remains very much underexplored in supramolecular coordination cages. Herein, we show that encapsulation of small aromatic aldehydes inside a supramolecular coordination cage allows the reduction of these aldehydes with the mild reducing agent sodium cyanoborohydride to proceed with high selectivity (ketones and esters are not reduced) and in good yields. In the absence of the cage, low pH conditions are essential for any appreciable conversion of the aldehydes to the alcohols. In contrast, the specific microenvironment inside the cage allows this reaction to proceed in bulk solution that is pH-neutral, or even basic. We propose that the cage acts to stabilise the protonated oxocarbenium ion reaction intermediates (enhancing aldehyde reactivity) whilst simultaneously favouring the encapsulation and reduction of smaller aldehydes (which fit more easily inside the cage). Such dual action (enhancement of reactivity and size-selectivity) is reminiscent of the mode of operation of natural enzymes and highlights the tremendous promise of cage architectures as selective catalysts.

## Abstract 2

Herein, we use a supramolecular coordination cage as a catalyst for the reduction of aldehydes to the corresponding alcohols using a weak hydride donor in neutral water, with a mode of action reminiscent of natural enzymes.

## Graphical abstract

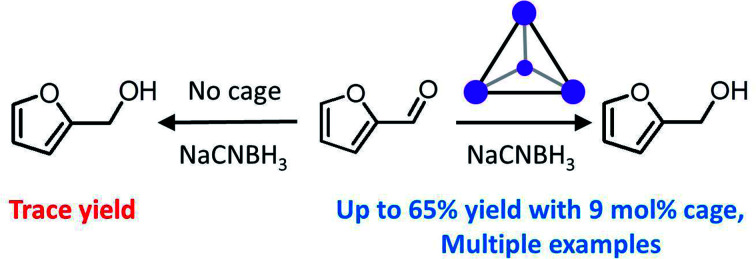

## Introduction

Supramolecular coordination cages fascinate chemists on account of their ability to enforce well-defined microenvironments on species hosted in their cavities.^1^ This has led to applications in areas such as molecular recognition,^2^ catalysis,^3^ resolutions and separations,^4^ and the stabilisation of otherwise unstable species,^5^ to name but a few. However, perhaps the most promising area of application of such cages is their potential to accelerate organic transformations.^6^

The potential for enhanced or altered reactivity inside cages is very well exemplified by the work of Raymond, Bergmann and co-workers using assemblies of the type M_4_L_6_ (M = Ga^III^, Al^III^, In^III^, Fe^III^, Ti^IV^, or Ge^IV^, and L = *N*,*N*′-bis(2,3-dihydroxybenzoyl)-1,5-diaminonaphthalene).^7^ These cages have been shown to facilitate the formation of (and stabilise) hydrolysis-prone species such as iminium and phosphonium cations in water,^8^ and also to give rise to dramatically increased p*K*_a_ values for protonated amines bound within their cavities.^9^ Moreover, these cages have been used to promote a number of catalytic reactions, such as the hydrolysis of orthoformates,^10^ acetal hydrolysis,^11^ Nazarov cyclisations,^12^ terpene cyclisations,^13^ and Prins reactions.^14^ Such observations have led Raymond and his colleagues to propose that the underlying cause of the enhanced reactivity in the above-named reactions is related to the ability of the cage to stabilise positively-charged transition states, possibly through interaction of the aromatic units (in the ligands forming the edges of the cage) with the carbocations that develop in the substrates during these reactions.^15^

Our initial interest in such cages stemmed from our ongoing attempts to up-grade furan derivatives to higher value products by electrosynthesis.^16^ For example, the furan derivative furfural (furan-2-carbaldehyde) is a major renewable chemical feedstock, the controlled (electro)reduction of which can yield furfuryl alcohol and 2-methylfuran, which are precursor chemicals for the sustainable production of polymers and fuels.^17,18^ However, the electroreduction of furfural can also lead to other products of somewhat lower value, including dimeric and polymeric species.^19,20^ Encapsulation of furfural inside a small supramolecular cage might prevent the oligomerisation of reactive intermediates during (electro)reduction and hence favour the production of furfuryl alcohol and/or 2-methylfuran. However, before such a hypothesis can be tested, it is first necessary to establish whether (and how) furfural binds within a given cage, and how this might be expected to affect its reactivity.

Amongst the numerous diverse coordination cage architectures that have been reported to date, the anionic tetrahedral Fe^II^_4_L_6_ iminopyridine complex (where L is the bis-imine product resulting from the reaction between 4,4′-diaminobiphenyl-2,2′-disulfonic acid and 2-formylpyridine) reported by Nitschke and co-workers in 2008 seemed to be an excellent first choice of cage for these purposes on account of its ease of synthesis (self-assembling in aqueous solution from commercial reagents) and its amenability to interrogation by solution-phase NMR spectroscopy.^21^ Moreover, Nitschke and co-workers have previously shown that furan binds inside this cage with *K*_a_ = (8.3 ± 0.7) × 10^3^ and a rate constant for uptake of 2.1 ± 0.3 M^−1^ s^−1^ at 298 K,^22^ meaning that encapsulation of furan by the cage is essentially quantitative after equilibration overnight at 50 °C. It seemed to us likely, therefore, that furfural would be similarly readily encapsulated. These Fe^II^_4_L_6_ cages have been the subject of fairly intense study over the past 10 years or so,^23^ but the potential for catalytic activity with these cages remains somewhat underexplored, with only a few examples reported to date.^6*r*,24^ Hence a study of the encapsulation and reactivity of furfural within these cages appeared to be warranted.

**Scheme 1:**
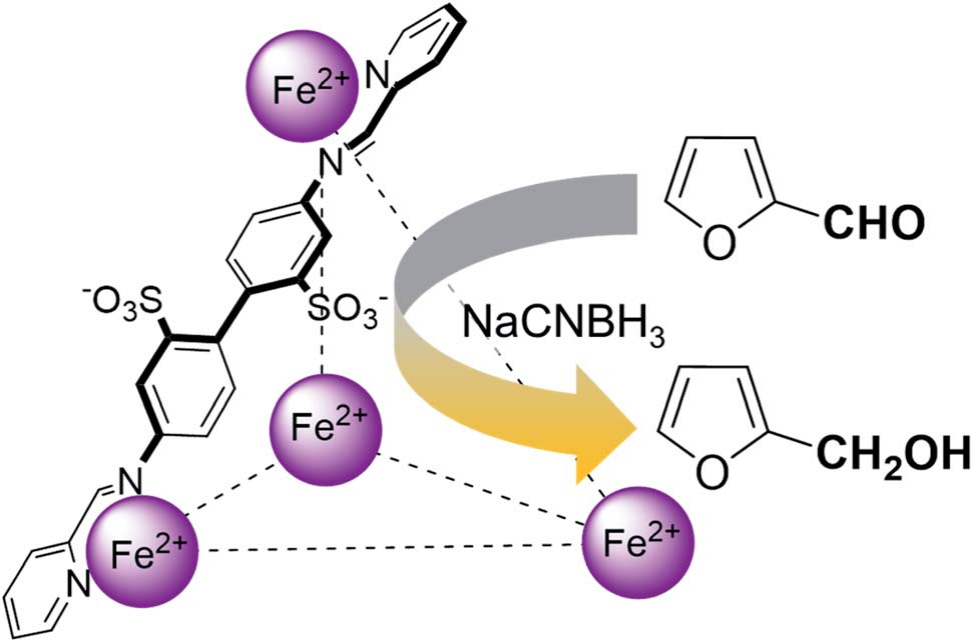
An illustration of the general class of reactions explored in this work, using the example of furfural reduction to furfuryl alcohol using sodium cyanoborohydride (NaCNBH_3_) as the reducing agent. Only one of the six identical edges of the anionic Fe^II^_4_L_6_ cage is shown for clarity.

Herein, we show that the Fe^II^_4_L_6_ cage does indeed bind furfural, and that (simply as a function of this binding) the reactivity of the encapsulated furfural is dramatically altered. Specifically, we demonstrate that the Fe^II^_4_L_6_ cage is in fact a general catalyst for the (non-electrochemical) reduction of a range of aromatic aldehydes to their corresponding alcohols using the weak hydride donor sodium cyanoborohydride (see Scheme 1). Using a range of control and competition reactions, we show that the Fe^II^_4_L_6_ cage architecture is essential for this enhanced conversion and that the cage is a genuine catalyst for the reduction of these carbonyls to their corresponding alcohols under our very mild conditions. To the best of our knowledge, only one example of the hydrogenation of aldehydes to the corresponding alcohols in a supramolecular coordination cage has yet been reported (very recently), requiring the use of strongly electron-withdrawing groups on the aldehyde substrate.^25^ Therefore, the work reported herein constitutes the first general demonstration of the conversion of non-activated aldehydes to their corresponding alcohols inside a supramolecular coordination cage.

## Results and discussion

**Fig. 1:**
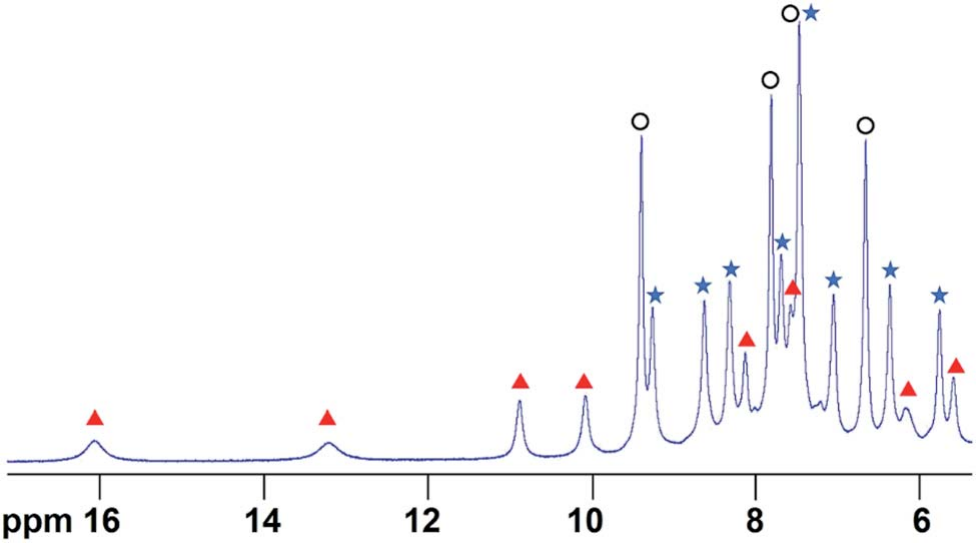
The ^1^H NMR spectrum of the Fe_4_L_6_ cage in D_2_O in the presence of 10 equivalents of furfural after heating to 50 °C for 1 h. Peaks labelled with blue filled stars indicate free cage, peaks with black open circles indicate free furfural and peaks denoted with red triangles constitute a new set of cage-like peaks that all show exchange cross-peaks to corresponding free cage peaks (see Fig. S1†). COSY and NOESY NMR data indicate that the peak at 7.4 ppm consists of signals emanating from both free cage and free furfural that are coincident.

In order to determine the extent of any encapsulation of furfural by the Fe^II^_4_L_6_ cage, ^1^H NMR spectroscopy was used to monitor the changes that occur upon incubation of the cage with 10 equivalents of furfural at 50 °C for 1 h in D_2_O (Fig. 1). Although it was not possible to assign peaks specifically to encapsulated furfural in this spectrum, NOESY NMR spectroscopy (see Fig. S1 in the ESI†) revealed a number of cross-peaks between this new set of peaks and those corresponding to free cage, which were attributed to the dynamic exchange between free cage and cage containing furfural. With these assumptions, a literature method^26^ was used to determine the binding constant of furfural inside the cage as *K*_a_ = 1.0 × 10^3^ M.

**Fig. 2:**
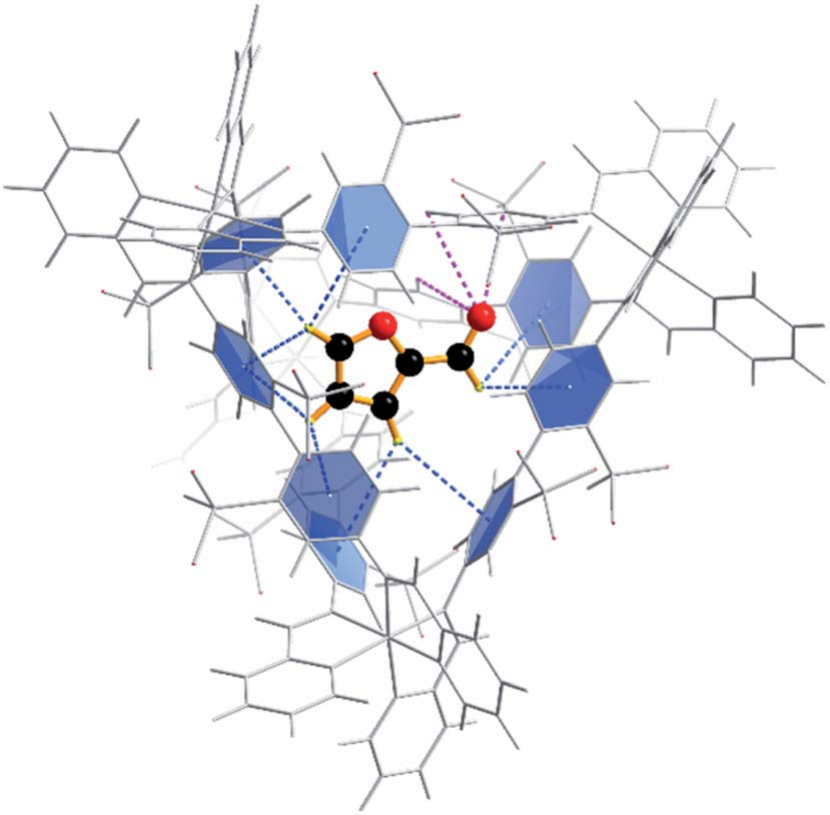
Calculated orientation of the furfural substrate within the Fe_4_L_6_ cage. Dashed lines depict the two hydrogen bond types that anchor the substrate in the cage: CH⋯π interactions with the aromatic rings of the cage ligands (blue); and O⋯H–C interactions with the carbonyl group projected towards a triangular opening in the wall of the cage (magenta).

To confirm that furfural can indeed reside within the cage, we next explored the energetics of furfural encapsulation by computational methods (see also ESI†).^27^ The resulting optimised structure shows that there is ample room for furfural to bind within the cage, and that when it does so it is anchored primarily by CH⋯π hydrogen bonds from the hydrogens on the furan ring to the aromatic rings of the cage ligands (Fig. 2).^28^ Several interactions can be identified, ranging between 3.1 and 3.3 Å. In addition, there are three close contacts between the carbonyl oxygen and protons lining one of the triangular openings of the tetrahedral cage. This combination of non-covalent interactions orientates the substrate within the cage as shown in Fig. 2.

**Fig. 3:**
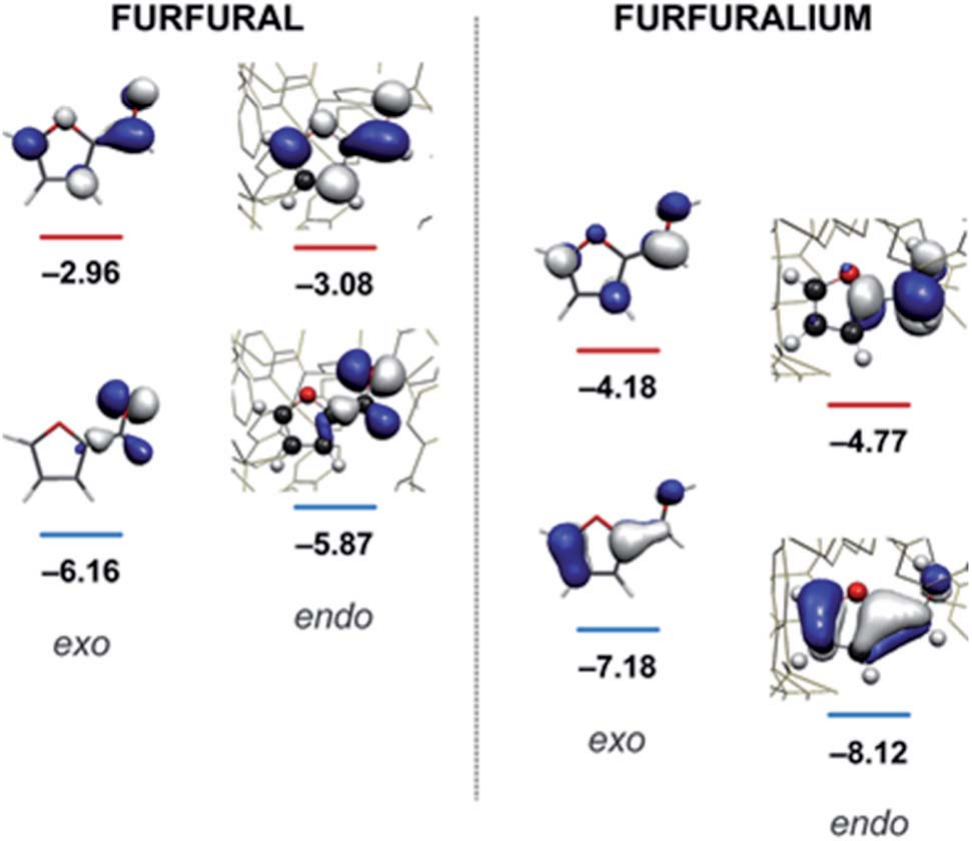
Calculated HOMO and LUMO energies (eV) for furfural (left) and the protonated furfuralium form (right), outside (exo) and inside (endo) the Fe_4_L_6_ cage.

Quantum mechanical calculations on the optimised structure at the BP86 level of theory showed that encapsulated furfural is 81.5 kcal mol^−1^ more stable than exohedral (*i.e.* free) furfural. The hydrogen-bonds to the carbonyl moiety are weak and are a product of its position (projected towards the triangular face). The orientation is therefore dictated by the CH⋯π hydrogen bonds between C–H bonds (from the aromatic R-group of the aldehyde) and the cage ligands. Moreover, the calculations suggest that the lowest unoccupied orbital (LUMO) of furfural is stabilised relative to the highest occupied orbital (HOMO) upon encapsulation: furfural inside the Fe_4_L_6_ cage experiences a 9.5 kcal mol^−1^ stabilisation of the LUMO compared to the substrate outside the cage (Fig. 3). Such a lowering of the LUMO energy is most intriguing, as it suggests that nucleophilic attack at the carbonyl carbon will be facilitated upon encapsulation, relative to the situation for furfural in free solution.

In their study of monoterpene-like cyclisation reactions using analogous M_4_L_6_ cages to those that are employed here, Toste, Bergmann, Raymond and co-workers postulated that the M_4_L_6_ cage was acting to stabilise the protonated aldehyde oxocarbenium ions in their substrates, leading to enhanced reactivity for cyclisation.^14^ As shown in Fig. 3 (see also ESI†), calculations suggest that the protonated oxocarbenium ion of furfural (“furfuralium”) can also be accommodated by the cage. The effect of protonation of the furfural in this way is also to lower the LUMO (right hand side of Fig. 3) and hence render the carbonyl moiety easier to reduce. There are distinct parallels here with acid catalysis of carbonyl reduction in bulk solution, where the effect of protonation is to withdraw electron density from the carbonyl moiety, rendering nucleophilic attack more facile. Indeed, reductions of aldehydes to the corresponding alcohols by the mild reducing agent sodium cyanoborohydride are generally held to work effectively only in acidic solutions.^29^ This suggested to us that if the aldehyde was indeed encapsulated by the anionic Fe_4_L_6_ cage, and if such cages were indeed capable of stabilising protonated substrates (which might normally only form to a significant degree in acid solution), then reactions that would otherwise require acidic conditions might occur in the presence of cage, even though the medium outside the cage might be neutral (or even basic).

To explore this hypothesis, we therefore studied the reduction of furfural to furfuryl alcohol using the mild reducing agent NaCNBH_3_ both in the presence and absence of cage. A typical procedure is given in the Experimental section. The cage was prepared as the tetramethylammonium salt as described by Nitschke and co-workers,^21^ and was isolated in a pure form prior to use for the following experiments. The results (see Table 1, entry 1), indicate that after extraction of the reaction mixture into organic solvent and purification by column chromatography, a 65% isolated yield of furfuryl alcohol is achieved after a reaction time of 6 h at 50 °C in the presence of 9 mol% of the cage at pH 7 (pH of bulk solution), whereas the yield under otherwise identical conditions but in the absence of cage gives only 4% furfuryl alcohol (no conversion to the alcohol is observed in the absence of a hydride source). This suggests that the cage is turning over between 6 and 7 times during the course of this reaction. Between 10 and 15% of the furfural starting material could be recovered after 6 h of reaction in the presence of cage. The remaining 20% or so of furfural that was neither converted to furfuryl alcohol nor recovered unchanged is probably consumed in reaction with the imine ligands of the cage, as suggested by LCMS analysis of the aqueous (cage-containing) phase after reaction (see ESI, Fig. S9†). Competitive inhibition by the furfuryl alcohol product does not appear to be a contributor to the less than quantitative conversion of furfural in this case: Fig. S10 (ESI†) suggests that furfuryl alcohol binds very weakly inside the cage under these conditions, and so should be readily displaced by furfural.

**Table 1:** Isolated yields of various alcohols obtained by the reduction of their corresponding aldehydes with NaCNBH_3_ in the presence and absence of 9 mol% cage

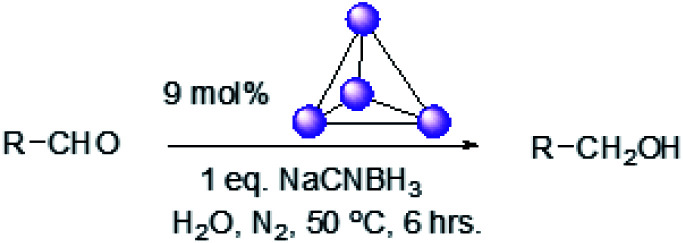				
Entry	Substrate	Product	Yield (9 mol% cage)	Yield (no cage)
1	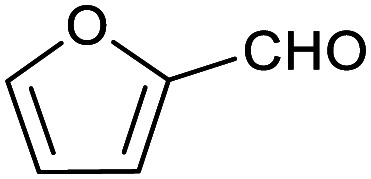	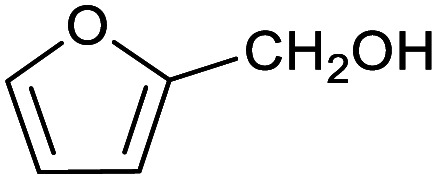	65 ± 1%	4 ± 1%
2	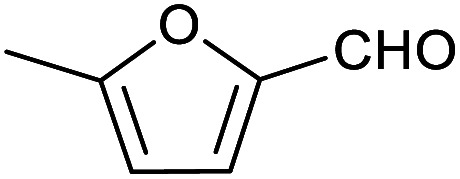	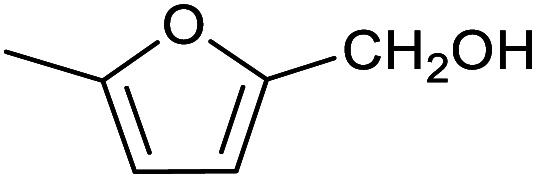	51 ± 2%	3 ± 1%
3	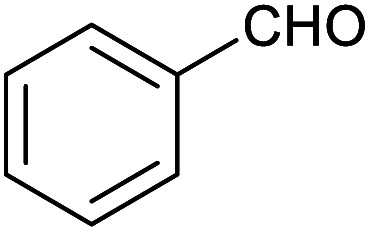	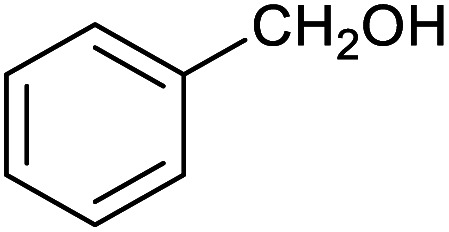	62 ± 4%	7 ± 2%
4	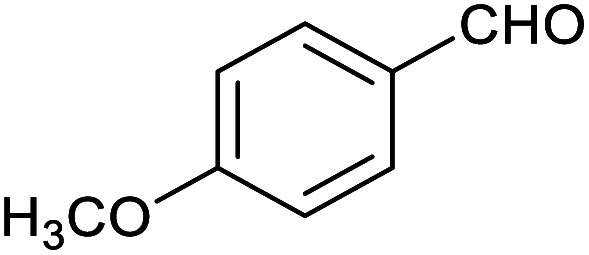	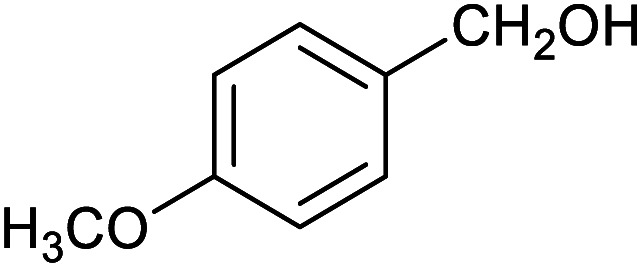	51 ± 1%	4 ± 1%
5	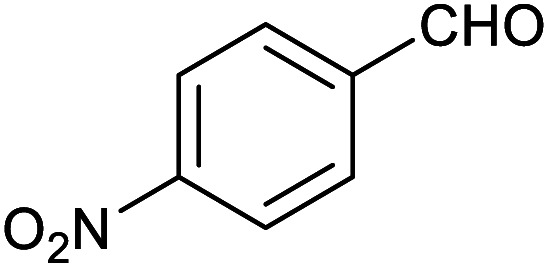	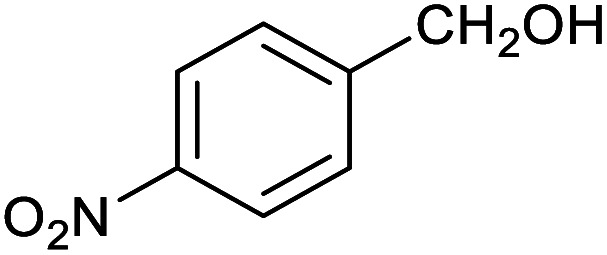	60 ± 5%	24 ± 2%
6	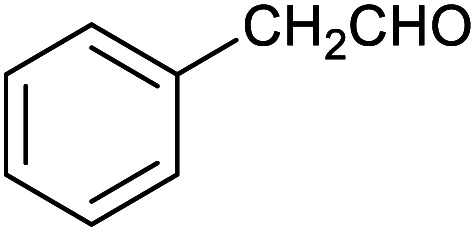	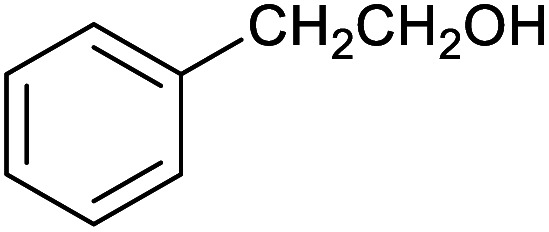	43 ± 3%	6 ± 1%
7	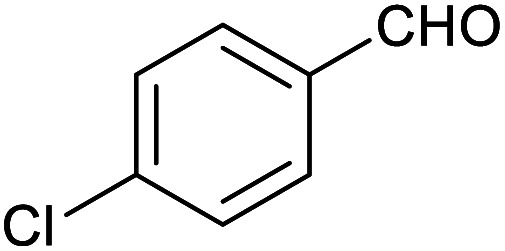	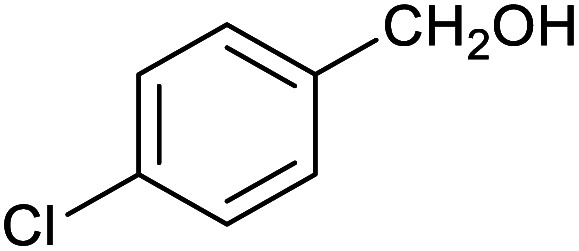	52 ± 5%	7 ± 1%
8	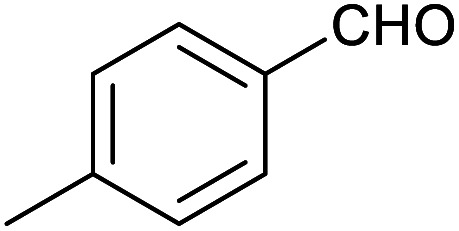	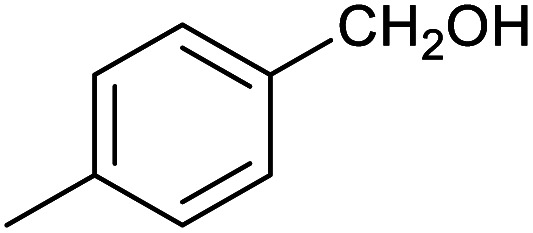	39 ± 3%	2 ± 1%
9	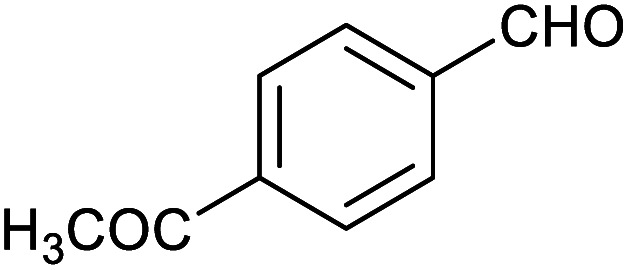	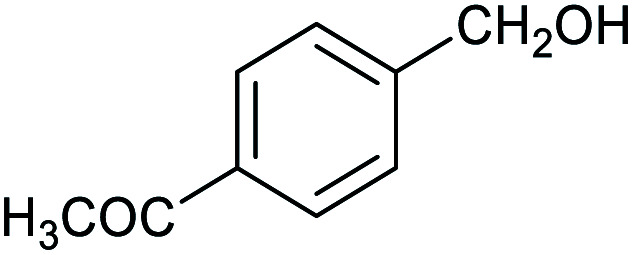	60 ± 2%	10 ± 2%
10	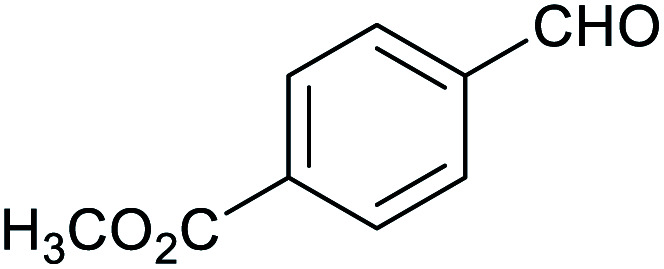	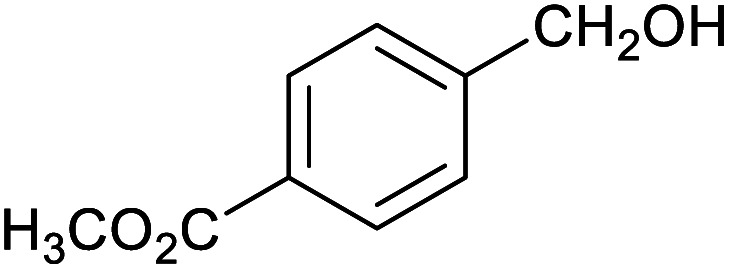	63 ± 2%	12 ± 1%
11	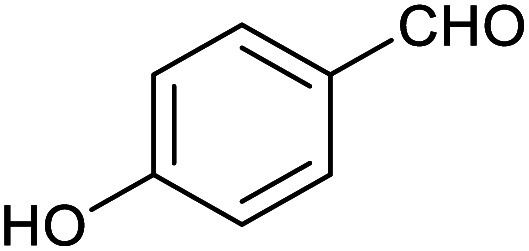	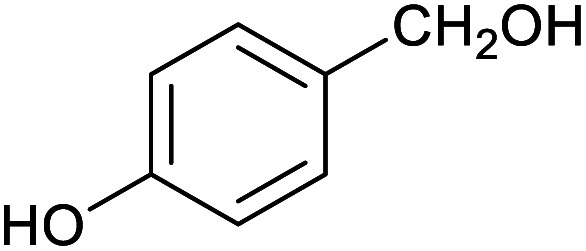	66 ± 2%	6 ± 1%
12	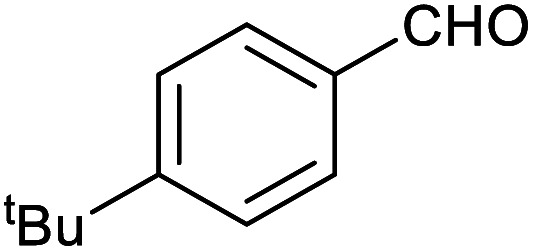	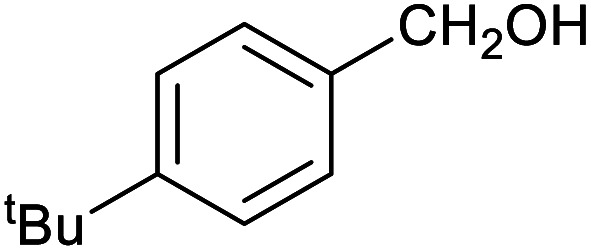	27 ± 1%	7 ± 1%

**Fig. 4:**
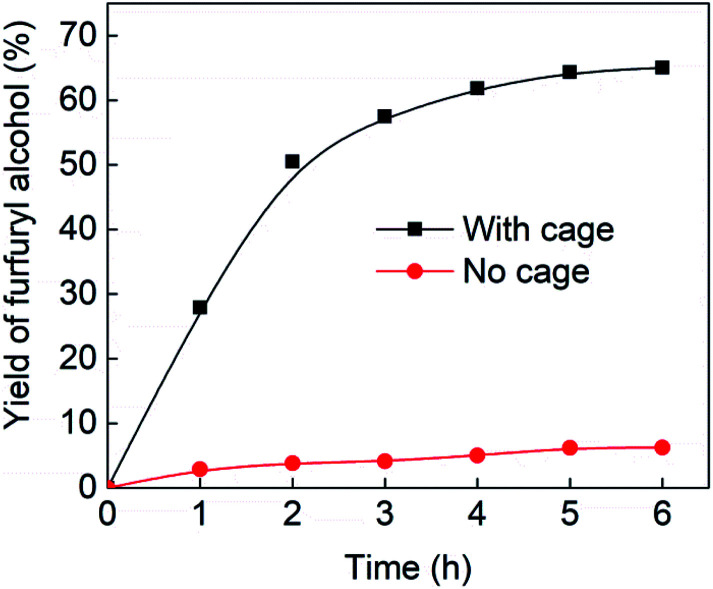
Yields of furfuryl alcohol *vs.* time in the presence of 9 mol% cage (black line and squares) or in the absence of cage (red line and circles). Yields in the presence of cage are isolated yields. Yields in the absence of cage were determined by ^1^H NMR and are likely to be slight overestimates of the amount of furfuryl alcohol produced.

Fig. 4 (black line) shows how the isolated yield of furfuryl alcohol varies with reaction time (again in the presence of 1 equivalent of NaCNBH_3_ relative to furfural at 50 °C, and using 9 mol% of the cage). These data can be compared to those obtained under otherwise identical conditions but in the absence of cage (red line and circles), and a comparison of the initial rates of reaction suggests a 10-fold acceleration of the rate of reaction in the presence of cage. The data in Fig. 4 were obtained by stopping a standard reaction procedure (see Experimental section) after the time periods indicated and extracting the reaction mixture as per the standard procedure. *In situ* monitoring of reaction progress by ^1^H NMR tended to give less reliable data, as certain cage peaks overlap with those of the products. Moreover, Fig. S11 (ESI†) shows that adding a further equivalent of both furfural and NaCNBH_3_ to an ongoing catalytic reaction at *t* = 6 h leads to an additional two catalytic turnovers of the cage. As competitive inhibition by the product is minimal in this case (see above), we attribute the drop-off in yields during this second cycle to cage decomposition through the pathways suggested in Fig. S9.† Alternatively, the Fe_4_L_6_ cage can be recovered from the aqueous phase after a single catalytic run (see Fig. S11 and associated discussion in the ESI†) and can then be re-used in catalytic experiments (albeit delivering lower conversion rates compared to fresh cage, most probably due to some decomposition of the cage). Taken together, these data suggest that the cage can, at least to some extent, be recycled and re-used for more than one catalytic reaction, preforming multiple turnovers in each experiment.

A further set of controls was undertaken in order to show that the specific supramolecular architecture of the cage is essential for aldehyde reduction, and that the catalysis is not mediated by the subcomponents of the cage. Hence, when the simple salt FeSO_4_ (0.36 equiv. relative to furfural) was used in place of the cage, the conversion of furfural to furfuryl alcohol was only ∼5% (the same as for the reaction in the absence of cage; Table 1, entry 1). Meanwhile, if a complex mimicking a single vertex of the cage (reported previously by Salles *et al.*^24*a*^) was used in place of the cage, then the yield of furfural alcohol after 6 h at 50 °C was only 10%, even in the presence of 40 mol% of this vertex complex. A competitive inhibition study was also undertaken using benzene, which Nitschke and co-workers have previously shown to be an excellent guest for this cage.^5*c*^ Hence, 0.09 equivalents of cage were incubated in D_2_O for an hour with 1 equivalent of benzene. After this time, 1 equivalent of furfural was added and the incubation continued for a further hour. Finally, one equivalent of NaCNBH_3_ was added and the reaction stirred at 50 °C for 2 hours. The yield of furfuryl alcohol from this reaction was found to be only 25–30%, whereas an experiment run under otherwise identical conditions but without an initial incubation of the cage with benzene typically yielded ∼50% furfuryl alcohol over the same time period (Fig. 4). Hence the presence of a competing guest for the cage does indeed seem to retard the reduction of furfural, further suggesting that the catalysis is occurring inside the cage.

Table 1 then shows that catalysis of aromatic aldehyde hydrogenation with this cage appears to be a general phenomenon; eleven further aldehydes as listed convert to their corresponding alcohols significantly more rapidly when using the cage as a catalyst compared to when no cage is present. The data in Table 1 suggest that increased steric bulk leads to poorer conversion of the aldehyde to its corresponding alcohol (compare entries 1 and 2, and entries 3, 6 and 8), which is consistent with increased sterics disfavouring encapsulation within the cage. Indeed, the length of *p-tert*-butylbenzaldehyde (entry 12) exceeds the interior dimension of the cage and shows considerably lower yields compared to the other aldehydes employed. DFT calculations (ESI, Fig. S8†), suggest that although an entire molecule of *p-tert*-butylbenzaldehyde cannot fit inside the cage, it is possible for the aldehyde group on this molecule to poke into the cage cavity through one of the triangular openings in the wall of the cage. This would account for the fact that conversion of *p-tert*-butylbenzaldehyde to its corresponding alcohol is enhanced by the cage, but to a much lesser extent than for the smaller aldehydes that are a better fit within the cage cavity. It is also noteworthy that conversions of all the aldehydes mentioned in Table 1 occur much faster at slightly elevated temperatures (50 °C) than they do at room temperature. For example, under identical conditions to those reported in Table 1, but at room temperature (298 K), the yield of 4-methylbenzyl alcohol (from the reduction of *p*-tolualdehyde) was 2% (±1%), whilst the yield of methyl 4-*tert*-butylbenzyl alcohol (from *p-tert*-butylbenzaldehyde) was 7% (±1%). This fact is also consistent with a mechanism whereby the improved flexibility (and perhaps also fluxionality) of the cage at elevated temperatures allows some of the bulkier compounds listed in Table 1 to encapsulate (or partially encapsulate) inside the cage, and hence convert more readily to their corresponding aldehydes. Experimental evidence for the interaction of *p-tert*-butylbenzaldehyde (the largest entry in Table 1 by volume) and *p*-tolualdehyde (entry 8, of intermediate volume between furfural and *p-tert*-butylbenzaldehyde) with the cage is provided by ^1^H NMR spectroscopy at 50 °C (ESI, Fig. S24 and S25†). In both cases, addition of the guest aldehyde to the cage leads to significant broadening of the ^1^H NMR signals corresponding to both the cage and the guest, consistent with an intermediate rate of exchange of the guest in and out of the cage on the NMR timescale as previously reported by Nitschke and co-workers for the binding of guests within a range of Fe^II^_4_L_6_ cages.^30^

Attempts at using still more bulky aldehydes (namely 9-anthracenecarboxaldehyde, 3,5-di-*tert*-butylbenzaldehyde, 4-(diphenylamino)benzaldehyde and 3,5-di-*tert*-butyl-2-hydroxybenzaldehyde, all which might have been expected to be completely excluded from the cage on steric grounds) were unsuccessful, as none of these aldehydes are water-soluble to any significant degree. Therefore, there was no conversion of these aldehydes in either the presence or absence of cage. In contrast, all the aldehydes shown in Table 1 exhibit at least partial water solubility, allowing these species to dissolve in bulk solution and thus gain access to the cage cavity. Electron-withdrawing substituents (entries 5, 9 and 10) tend to give rise to greater conversion to the alcohol than is evident with electron-donating substituents (entries 2, 4, 7 and 8). These results are consistent with nucleophilic hydride attack at the carbonyl carbon, and the same trend can be observed both with and without cage. However, the extent of conversion is always significantly better when cage is present.

In order to probe the selectivity of the cage further, a series of reactions were performed involving other potentially reducible chemical moieties, as well as mixtures of aldehydes. Hence, entries 9 and 10 in Table 1 show that when only one equivalent of NaCNBH_3_ is used, only the aldehyde is reduced and that there is no detectable competitive reduction of the ketone or ester moieties under these conditions. Meanwhile, a competition experiment between one equivalent of *p*-chlorobenzaldehyde and (much bulkier) *p-tert*-butylbenzaldehyde in the presence of only one equivalent of NaCNBH_3_ and 9 mol% cage leads to a 60% yield of *p*-chlorobenzyl alcohol and only a 13% yield of *p-tert*-butylbenzyl alcohol. This compares to a 7% yield of both alcohol products after 6 h when the same competition experiment is run in the absence of cage. These results suggest that the cage cavity provides a microenvironment that can bias relative product distributions away from those observed in the absence of cage. Moreover, the yield of *p-tert*-butylbenzyl alcohol is halved in this cage-containing competition reaction, relative to its value when *p-tert*-butylbenzaldehyde is reduced in the presence of cage but without any competitor substrate. The implication is that the cage not only enhances the extent of aldehyde reduction, but that it can also impose some selection on the reaction outcome by preferentially catalysing the reduction of those aldehydes that fit more easily inside the cage. Such size-selective catalysis is reminiscent of the mode of action of natural enzymes.

**Fig. 5:**
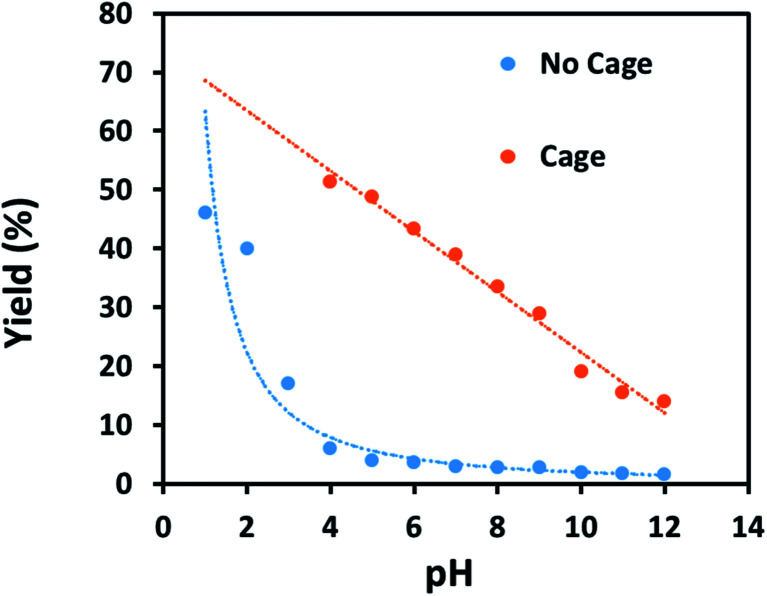
Yields of 4-methylbenzyl alcohol *vs.* pH in the presence of 9 mol% cage (orange points and line) or in the absence of cage (blue points and line). All yields are isolated yields after 6 h of reaction under the conditions reported in the Experimental section.

Finally, some direct evidence in support of stabilisation of protonated intermediates as the mechanism for the enhanced reactivity for aldehyde reduction in the presence of the anionic Fe_4_L_6_ cage was obtained. Fig. 5 shows the effect that altering the pH of the bulk solution has on the yield of 4-methylbenzyl alcohol (from tolualdehyde, both of which have methyl groups which are readily-discernable by ^1^H NMR spectroscopy, aiding analysis) under the standard reaction conditions reported in the Experimental section in the presence and absence of cage. When cage is present, a clear trend is observed whereby the yield increases in a linear fashion as the pH of the bulk solution is varied between 12 and 4 (pH lower than 4 was not probed as the cage is known to be unstable under acidic conditions^21^). This stands in contrast to the reaction yields in the absence of cage, which are essentially basal until pH 4, after which there is a marked increase in yield with each successive reduction in pH. The implication is that the cage is stabilising the protonated form of the aldehyde in the basic and near-neutral regime, effectively increasing the basicity of the encapsulated substrate by around 5 p*K*_a_ units under neutral conditions (compare the yields obtained with and without cage at pH 7 and pH 2 respectively). Again, alteration of substrate basicities as a function of binding in order to enhance a reaction that would otherwise not take place in bulk solution is a strategy often employed by enzymes.

## Conclusions

In summary, we have shown that a variety of aromatic aldehyde substrates can be reduced to their corresponding alcohols in good yields using the mild reducing agent NaCNBH_3_ as a hydride source and using Nitschke's Fe_4_L_6_ cage as an enzyme-like catalyst. In the absence of cage, reduction of the aldehydes is limited. ^1^H and NOESY NMR spectroscopy, DFT calculations, control reactions with sub-components of the cage and competition reactions all suggest that catalysis occurs inside the cage. Complete selectivity for aldehyde reduction (over the reduction of ketones and esters) is observed. Meanwhile, computational analysis and pH-dependency studies suggest that the reason for the enhanced reactivity in the presence of cage is the stabilisation of protonated oxocarbenium ions inside the cage, which activates the encapsulated species to nucleophilic attack. Work to expand the scope of these studies (in particular, target reactions, type of cage catalyst and alternative reaction conditions) is currently ongoing in our laboratories.

## Experimental section

### Typical procedure

100 mg (0.027 mmol, 0.09 equiv.) of the [Fe_4_L_6_] cage^21^ (as the tetramethylammonium salt) was weighed into a 14 mL vial with a small magnetic stir-bar. The vial was closed with a rubber septum and kept under nitrogen using Schlenk techniques. Aldehyde (0.3 mmol, 1 equiv.) was added to the same vial (using a micro-syringe for liquids) under a nitrogen atmosphere. 3 mL of degassed distilled water was then injected into the same vial under nitrogen, and the reaction mixture stirred for 1 h at 50 °C. Meanwhile, NaCNBH_3_ (0.3 mmol, 19 mg, 1 equiv.) was weighed out inside a glove-box into a separate vial sealed with a rubber septum. 2 mL of degassed distilled water was then injected into this vial containing the NaCNBH_3_ under nitrogen. The aqueous solution of NaCNBH_3_ was then transferred to the main reaction vial under nitrogen. The reaction mixture was then kept stirring for another 6 hours at 50 °C inside the sealed vial. After this time, the reaction mixture was allowed to cool down to room temperature before extraction of the products with dichloromethane (4 × 20 mL). The organic layers were combined and dried over MgSO_4_. The solvent was removed under reduced pressure and the products were isolated by column chromatography using diethyl ether/hexane mixtures as the eluents (the ratio varied with the *R*_f_ values of the product; typically, 20–40% diethyl ether in hexane was used). The solvents were then carefully removed under reduced pressure at 25 °C and finally the product was dried under high-vacuum with cooling (in order to prevent any evaporation of the products). Characterisation of all alcohol products is given in the ESI.† Control reactions without cage were conducted in an entirely analogous manner, save for the addition of cage. Under these standard conditions, the pH of the reaction medium was 7. The pH could be adjusted to other values by using sodium bicarbonate and/or NaOH (to move more basic), or HCl or phosphoric acid (to move to more acidic pH).

## Author contributions

AP, MAS and DYO performed the experiments and analysed the data. SS performed the calculations. MDS conceived the project and analysed experimental data. All authors contributed to the writing of the manuscript.

## Conflicts of interest

There are no conflicts of interest to declare.

## Supplementary Material

SC-012-D1SC00896J-s001

## References

[cit1] (k) Fujita M. and Yoshizawa M., in Modern Supramolecular Chemistry, ed. F. Diederich, P. J. Stang and R. R. Tykwinski, Wiley-VCH, Weinheim, 2008, pp. 277–313

[cit2] Bloch W. M., Abe Y., Wandtke C. M., Dittrich B., Clever G. H. (2016). J. Am. Chem. Soc..

[cit3] Zhang D., Martinez A., Dutasta J.-P. (2017). Chem. Rev..

[cit4] Luo D., Zhou X.-P., Li D. (2015). Angew. Chem., Int. Ed..

[cit5] Iwasawa T., Hooley R. J., Rebek J. (2007). Science.

[cit6] Yoshizawa M., Tamura M., Fujita M. (2006). Science.

[cit7] Caulder D. L., Powers R. E., Parac T. N., Raymond K. N. (1998). Angew. Chem., Int. Ed..

[cit8] Ziegler M., Brumaghim J. L., Raymond K. N. (2000). Angew. Chem., Int. Ed..

[cit9] Pluth M. D., Bergman R. G., Raymond K. N. (2007). J. Am. Chem. Soc..

[cit10] Hong C. M., Bergman R. G., Raymond K. N., Toste F. D. (2018). Acc. Chem. Res..

[cit11] Pluth M. D., Bergman R. G., Raymond K. N. (2007). Science.

[cit12] Pluth M. D., Bergman R. G., Raymond K. N. (2007). Angew. Chem., Int. Ed..

[cit13] Hastings C. J., Pluth M. D., Bergman R. G., Raymond K. N. (2010). J. Am. Chem. Soc..

[cit14] Hart-Cooper W. M., Clary K. N., Toste F. D., Bergman R. G., Raymond K. N. (2012). J. Am. Chem. Soc..

[cit15] Pluth M. D., Bergman R. G., Raymond K. N. (2009). Acc. Chem. Res..

[cit16] Shipman M. A., Sproules S., Wilson C., Symes M. D. (2019). R. Soc. Open Sci..

[cit17] Dalvand K., Rubin J., Gunukula S., Clayton Wheeler M., Hunt G. (2018). Biomass Bioenergy.

[cit18] Chadderdon X. H., Chadderdon D. J., Matthiesen J. E., Qiu Y., Carraher J. M., Tessonnier J.-P., Li W. (2017). J. Am. Chem. Soc..

[cit19] Kwon Y., Schouten K. J. P., van der Waal J. C., de Jong E., Koper M. T. M. (2016). ACS Catal..

[cit20] Hallal J. L. J., Lucho A. M. S., Gonçalves R. S. (2005). Mater. Res..

[cit21] Mal P., Schultz D., Beyeh K., Rissanen K., Nitschke J. R. (2008). Angew. Chem., Int. Ed..

[cit22] Smulders M. M. J., Nitschke J. R. (2012). Chem. Sci..

[cit23] Zhang D., Ronson T. K., Nitschke J. R. (2018). Acc. Chem. Res..

[cit24] Salles Jr A. G., Zarra S., Turner R. M., Nitschke J. R. (2013). J. Am. Chem. Soc..

[cit25] Morimoto M., Cao W., Bergman R. G., Raymond K. N., Toste F. D. (2021). J. Am. Chem. Soc..

[cit26] Hristova Y. R., Smulders M. M. J., Clegg J. K., Breiner B., Nitschke J. R. (2011). Chem. Sci..

[cit27] Thompson M., ArgusLab 4.01, PlanariaSoftware LLC, Seattle, WA, USA, 2005, see http://www.ArgusLab.com

[cit28] Nishio M. (2011). Phys. Chem. Chem. Phys..

[cit29] Borch R. F., Bernstein M. D., Durst H. D. (1971). J. Am. Chem. Soc..

[cit30] Ronson T. K., Meng W., Nitschke J. R. (2017). J. Am. Chem. Soc..

