# Supplementary material for: Selective aldehyde reductions in neutral water catalysed by encapsulation in a supramolecular cage
Source: Chem Sci. 2021 Mar 12;12(14):5082–90. doi: 10.1039/d1sc00896j (PMC8179549; doi:10.1039/d1sc00896j)
Supplement: SC-012-D1SC00896J-s001 [file SC-012-D1SC00896J-s001.pdf]

Electronic Supplementary Material (ESI) for Chemical Science.
This journal is © The Royal Society of Chemistry 2021

Electronic Supplementary Information

Selective Aldehyde Reductions in Neutral Water Catalysed by Encapsulation in a Supramolecular Cage

Avishek Paul,† Michael A. Shipman,† Dolapo Y. Onabule, Stephen Sproules and Mark D. Symes**

WestCHEM, School of Chemistry, University of Glasgow, University Avenue, Glasgow, G12 8QQ, United Kingdom

†These authors contributed equally to this work

**Email: mark.symes@glasgow.ac.uk; stephen.sproules@glasgow.ac.uk*

Index	*Page*
General experimental remarks	S3
Figure **S1**	S4
Details of computational calculations	S5
Figure **S9**	S30
Figure **S10**	S31
Figure **S11**	S32
Characterisation of isolated products	S33
Figure **S23**	S45
Figure **S24**	S46
References	S47

General Experimental Remarks: The $Fe^{II}_4L_6$ cage was prepared as previously reported by Nitschke and co-workers.[S1] Iron(II) sulfate heptahydrate (≥99%), furfural (99%), benzaldehyde (≥99%), and 5-methylfurfural (99%) were purchased from Sigma-Aldrich. 4,4′-diaminobiphenyl-2,2′-disulfonic acid (≥70%), tetramethylammonium hydroxide (98%), sodium cyanoborohydride (95%), dimethyl sulfoxide (99.8%), nitrobenzaldehyde (99%), anisaldehyde (99%), phenyl acetaldehyde (95%), acetophenone (99%) and chlorobenzaldehyde (99%) were purchased from Alfa Aesar. Hexane (≥95%), acid-washed sand and sodium bicarbonate (≥99.7%) were purchased from Fischer Scientific. Ethanol (100%), dichloromethane (100%), magnesium sulfate and acetone (≥95%) were purchased from VWR. Hydrochloric acid was purchased from Honeywell. Silica 60 (0.04-0.063 mm) was purchased from Merck. 4-(Diphenylamino)benzaldehyde was purchased from TCI. D_2O (99.9%) and $CDCl_3$ (99.8%) were purchased from Cambridge Isotope Laboratories. Pyridine-2-carboxaldehyde (99%) was purchased from Acros Organics. All chemical reagents and solvents were used as purchased. All ^1H and ^{13}C NMR spectra were recorded on a Bruker AVI 400 instrument or a Bruker AVIII 500 instrument (as indicated in the text), at a constant temperature of 298 K. Chemical shifts are reported in parts per million from low to high field. Coupling constants (*J*) are reported in hertz (Hz). Standard abbreviations indicating multiplicity were used as follows: m = multiplet, t = triplet, d = doublet, s = singlet, br = broad. LC-MS mass spectra (ESI, positive mode, Bruker micrOTOF-Q machine) were collected by the services facility at the School of Chemistry, University of Glasgow.

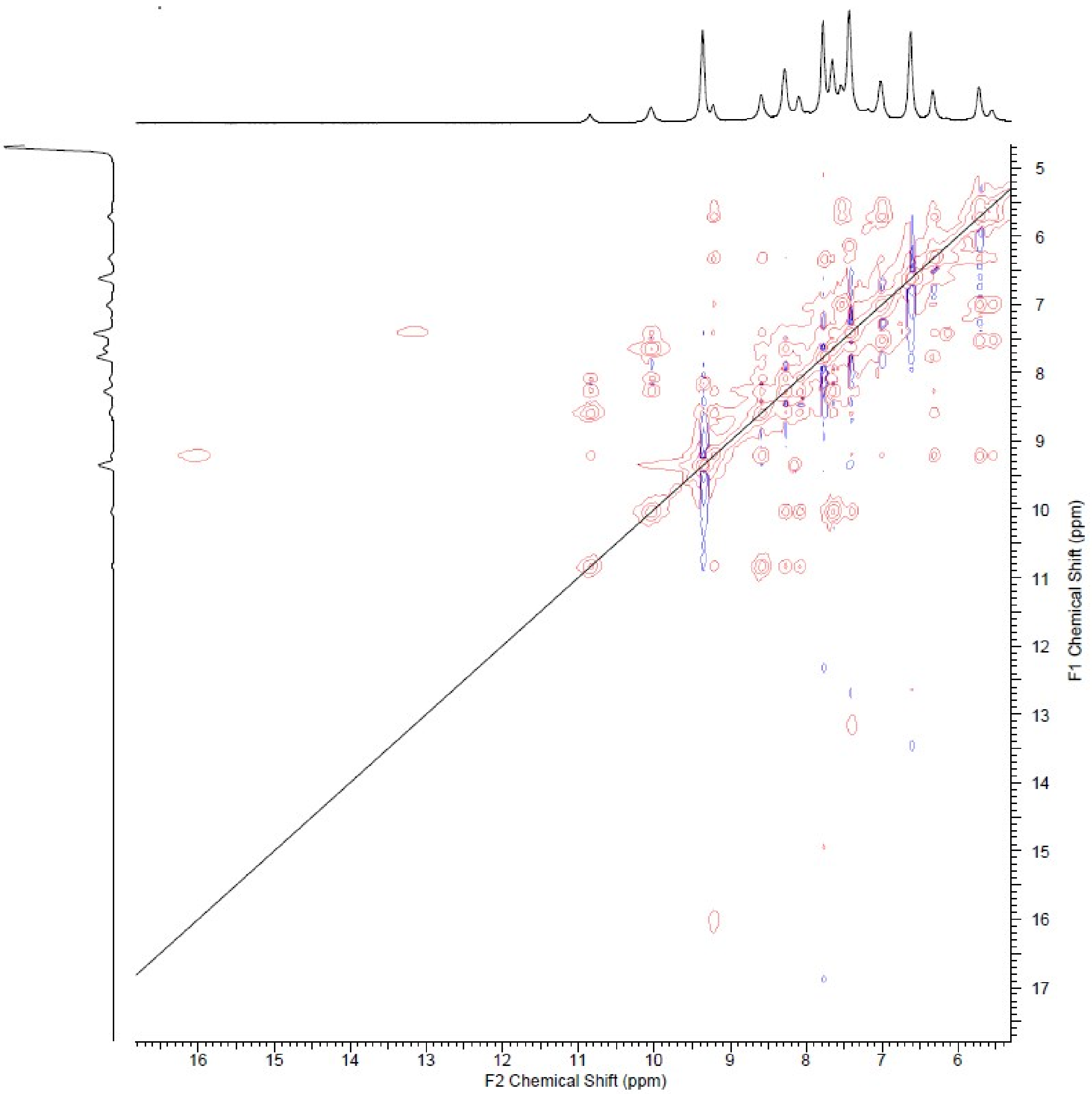

Figure S1: NOESY spectrum of the Fe_4L_6 cage in D_2O in the presence of 10 equivalents of furfural after heating to 50 °C for 1 h, showing exchange cross-peaks between peaks assigned to free cage and the new set of cage-like peaks that appears upon addition of furfural. Mixing time = 0.3 s, T = 298 K, 500 MHz.

Details of computational calculations

The program package ORCA was used for all calculations.[S2] The input geometry for all molecules were generated using ArgusLab beginning from crystallographic coordinates.[S3] Single point calculations on the generated structures were carried out at the BP86 level of theory.[S4] Calculations were performed in the gas phase and in an infinite continuum using the conductor-like screening model (COSMO).[S5] A segmented all-electron relativistically contracted basis set of triple-ζ quality (def2-TZVP) for C, H and O atoms of furfural substrate.[S6] The cage atoms were described by a split-valence basis sets with one set of polarization functions (def2-SVP),[S7] with Grimme's dispersion correction D3.[S8] A scalar relativistic correction was applied using the zeroth-order regular approximation (ZORA) method[S9] as implemented by van Wüllen.[S10] The RI approximation combined with the appropriate Ahlrichs auxiliary basis set was used to speed up the calculations.[S11] The self-consistent field calculations were tightly converged (1×10^{-8} E_h in energy, 1×10^{-7} E_h in the density charge, and 1×10^{-7} in the maximum element of the DIIS[S12] error vector). The geometry was converged with the following convergence criteria: change in energy $<10^{-5}$ E_h, average force $<5 \times 10^{-4}$ E_h Bohr^{-1}, and the maximum force 10^{-4} E_h Bohr^{-1}. Canonical orbitals were generated with the program Molekel.[S13]

Table S1. Optimized Coordinates for Furfural@Fe_4

O	26.103496	1.982647	7.970626
C	25.014022	1.982235	8.600488
C	24.569007	0.784956	9.308091
O	25.303194	-0.357143	9.378099
C	23.376906	0.686812	9.985882
C	24.518349	-1.179320	10.125756
C	23.338593	-0.580359	10.490723
H	24.405118	2.874974	8.619591
H	24.799722	-2.186001	10.398557
H	22.537962	-1.029399	11.064622
H	22.617684	1.454163	10.096674
Fe	29.529320	5.145010	7.752050
Fe	16.657645	4.240447	9.061381
Fe	23.269656	-4.943525	2.693838
Fe	24.451656	-3.836448	15.612484
S	24.232595	5.810595	10.777608
S	30.294603	-0.214082	10.787883
S	26.002374	2.680028	3.414229
S	16.803383	-1.159678	6.252870
S	20.141554	3.093404	13.904829
S	25.965752	-6.265770	10.198073
S	22.171554	8.184955	8.283488
S	27.514705	1.318093	14.563280
S	28.217439	-0.503189	2.210755
S	20.001908	0.181186	2.532813
S	18.979970	-1.650650	14.038904
S	21.618683	-6.402377	8.349955
O	22.953396	6.845756	11.303112
O	30.176617	-1.582523	9.740740
O	23.932187	4.438757	11.426931
O	25.460520	6.266572	11.601614
O	25.911116	1.850450	2.113299
O	30.480760	-0.789568	12.211958
O	31.702657	0.371393	10.524639
O	15.317380	-0.728283	6.255951
O	24.373557	3.093042	3.808330
O	26.604969	3.995087	2.864616
O	19.147607	4.226441	14.255478
O	16.930907	-2.336938	7.510881
O	26.469710	-7.274728	11.257488
O	20.472340	2.321304	15.414995
O	16.870589	-1.952625	4.925885
O	21.415058	3.898698	13.555302
O	25.530888	-7.224666	9.064470
O	27.387929	-5.473436	9.621107
O	23.468311	8.536355	9.049046
O	27.833216	1.131892	16.251312
O	21.144659	9.562059	8.468714
O	26.368761	2.353611	14.478998
O	28.621494	0.989401	2.224815
O	22.641164	8.248522	6.810975
O	28.722985	2.121195	14.025434
O	29.361037	-1.151100	3.026048
O	21.158998	1.457143	2.397487
O	18.552981	-2.585798	15.427695
O	28.488319	-1.030586	0.587800
O	19.889273	-0.409438	1.107013
O	18.624425	0.872633	2.668791
O	21.896474	-7.325435	9.783952

O	17.914891	-0.529796	13.980295
O	18.597397	-2.530749	12.825897
O	21.057944	-7.421320	7.329370
O	20.368151	-5.540430	8.642529
N	29.488634	7.029006	8.591260
N	31.425229	4.769829	8.475933
N	16.297919	6.049937	8.141910
N	14.935567	3.378713	8.335786
N	22.831306	-4.234261	16.826444
N	27.655251	5.587747	6.838933
N	28.769847	4.583968	9.625558
N	29.712411	3.165731	7.019546
N	17.663938	3.831666	7.246622
N	16.937642	2.332983	9.908462
N	23.123922	-4.440380	14.102410
N	18.309224	5.159598	9.925528
N	26.115359	-3.626053	14.339770
N	23.678765	-2.964689	2.214143
N	21.510299	-4.592992	3.789440
N	23.887371	-1.811813	15.550660
N	24.579839	-4.948674	4.354603
N	15.736503	4.854756	10.804316
N	25.147263	-5.775316	15.753902
N	22.160100	-4.860810	0.954012
N	22.775524	-6.912212	3.037995
N	25.609703	-3.190636	17.192157
N	24.993210	-5.419507	1.668370
C	31.585873	5.915831	5.562272
C	29.818011	8.223354	8.003321
C	32.213928	5.593691	9.238757
C	15.535594	7.092025	8.605125
C	14.008312	3.939208	7.496850
C	22.739248	-4.078627	18.185630
C	31.896134	6.464469	4.315815
C	29.761435	9.396656	8.759366
C	33.503338	5.182052	9.583471
C	15.458210	8.268993	7.857259
C	12.862599	3.212824	7.163086
C	21.568478	-4.468048	18.840774
C	30.866660	6.939932	3.497706
C	29.379615	9.332429	10.104004
C	33.972721	3.942565	9.136556
C	16.158538	8.364846	6.649955
C	12.680267	1.930686	7.694539
C	20.514379	-5.016541	18.101513
C	29.538339	6.867701	3.927204
C	29.058599	8.102398	10.686964
C	33.156665	3.123467	8.350603
C	16.921948	7.286377	6.191751
C	13.639636	1.379321	8.550400
C	20.631709	-5.173424	16.716770
C	29.256984	6.310587	5.178790
C	29.122722	6.944287	9.907147
C	31.870976	3.561764	8.019624
C	16.980448	6.119632	6.960068
C	14.775160	2.129215	8.869252
C	21.815121	-4.778666	16.086777
C	27.879633	6.199720	5.696320
C	28.776472	5.608876	10.442530
C	30.933570	2.733557	7.233683
C	17.713307	4.917010	6.513434

C	15.879864	1.587945	9.693856
C	21.993897	-4.851640	14.618109
C	26.357024	5.431761	7.270430
C	28.403879	3.350093	10.078037
C	28.795179	2.347665	6.410587
C	18.186150	2.655087	6.778502
C	17.986361	1.857150	10.645377
C	23.328073	-4.457338	12.754810
C	25.977314	5.687622	8.596922
C	29.305209	2.276829	10.068409
C	27.953873	2.817946	5.392214
C	17.441039	1.472023	6.820161
C	18.540544	2.610102	11.689099
C	24.394392	-5.178441	12.208142
C	24.633112	5.632879	9.006036
C	29.013448	1.073236	10.731186
C	27.094275	1.956138	4.688013
C	17.912439	0.281527	6.250415
C	19.555233	2.090594	12.509108
C	24.578473	-5.272453	10.823638
C	23.626072	5.383198	8.052012
C	27.782945	0.930947	11.410684
C	27.099439	0.574104	4.987511
C	19.180385	0.255820	5.627066
C	20.035520	0.782418	12.284945
C	23.665586	-4.642258	9.953494
C	23.986182	5.123624	6.728175
C	26.867548	1.989609	11.387179
C	27.916850	0.099743	6.017895
C	19.914976	1.447353	5.549949
C	19.500553	0.035748	11.225736
C	22.564177	-3.961154	10.493435
C	25.326717	5.138219	6.345893
C	27.189120	3.189763	10.762541
C	28.754034	0.967893	6.716085
C	19.421967	2.631327	6.098995
C	18.474215	0.552862	10.434405
C	22.387191	-3.884008	11.877727
C	22.199101	5.361425	8.426758
C	27.443747	-0.270771	12.207882
C	26.261073	-0.396751	4.254807
C	19.778952	-1.001480	5.103723
C	21.020469	0.133768	13.186950
C	23.848695	-4.684598	8.485663
C	21.492517	6.550486	8.696051
C	27.185075	-0.184017	13.594085
C	26.596119	-0.843228	2.959290
C	20.243971	-1.125982	3.772578
C	20.678841	-1.012911	13.937082
C	22.985158	-5.425685	7.656800
C	20.198516	6.477057	9.223841
C	26.731428	-1.310097	14.286250
C	25.703622	-1.669111	2.264074
C	20.852355	-2.319797	3.355764
C	21.654130	-1.658541	14.701594
C	23.231918	-5.489241	6.278754
C	19.552389	5.234883	9.380818
C	26.518109	-2.528712	13.637977
C	24.542943	-2.163300	2.889334
C	20.990191	-3.408612	4.223658
C	22.977532	-1.210742	14.732158

C	24.302684	-4.807271	5.689674
C	20.217980	4.071207	8.983646
C	26.883765	-2.634791	12.282229
C	24.298888	-1.832849	4.224773
C	20.483788	-3.289794	5.531246
C	23.293090	-0.018263	14.053774
C	25.166060	-4.078362	6.537537
C	21.540746	4.129481	8.542762
C	27.324282	-1.515030	11.572600
C	25.136985	-0.936702	4.893405
C	19.912165	-2.096938	5.970301
C	22.328204	0.639821	13.281593
C	24.942027	-4.021646	7.912940
C	17.953320	5.862964	10.970041
C	26.814872	-4.732026	14.310226
C	23.262992	-2.683921	1.006625
C	20.884710	-5.723020	4.014291
C	24.436034	-1.149780	16.536510
C	25.812861	-5.123133	3.943483
C	16.583814	5.674847	11.502344
C	26.322634	-5.905941	15.064580
C	22.387750	-3.665110	0.324586
C	21.539186	-6.991326	3.618724
C	25.365152	-1.866184	17.442570
C	26.066878	-5.383917	2.512326
C	16.158401	6.257578	12.698913
C	26.990130	-7.133927	15.053840
C	21.778858	-3.399452	-0.904640
C	20.979035	-8.242394	3.891132
C	26.021855	-1.226355	18.497363
C	27.350247	-5.630837	2.015531
C	14.863441	5.996877	13.158231
C	26.445701	-8.209379	15.762580
C	20.947954	-4.369570	-1.474390
C	21.695557	-9.395127	3.552430
C	26.913209	-1.959455	19.287395
C	27.509970	-5.914819	0.655684
C	14.009429	5.164807	12.425442
C	25.248352	-8.053227	16.469364
C	20.736206	-5.588853	-0.820786
C	22.957366	-9.291383	2.955135
C	27.141900	-3.314092	19.020652
C	26.396179	-5.958194	-0.189733
C	14.459362	4.596064	11.231365
C	24.600518	-6.815739	16.460830
C	21.359708	-5.830134	0.405888
C	23.494198	-8.027793	2.696266
C	26.474172	-3.928792	17.958752
C	25.125870	-5.706175	0.333475
N	30.273990	5.842144	5.959132
O	20.661516	0.577918	8.387260
O	23.412719	1.538263	6.081353
O	22.627443	-1.799926	7.345380
H	32.383345	5.564975	6.203900
H	30.127774	8.265569	6.966898
H	31.855183	6.560453	9.567017
H	14.994300	7.011395	9.539502
H	14.154937	4.935127	7.096608
H	23.563939	-3.667616	18.754351
H	32.928629	6.526928	3.987145
H	30.015768	10.350532	8.308280

H	34.138248	5.821880	10.188254
H	14.860742	9.103141	8.211079
H	12.118335	3.640555	6.498898
H	21.478925	-4.350342	19.916033
H	31.100243	7.369849	2.528753
H	29.333107	10.240485	10.696755
H	34.973449	3.615752	9.400426
H	16.103316	9.276564	6.063525
H	11.794084	1.359487	7.436694
H	19.601302	-5.319139	18.604351
H	28.740509	7.240216	3.291540
H	28.760711	8.055033	11.730202
H	33.520456	2.158386	8.010963
H	17.453500	7.358381	5.247531
H	13.505289	0.377300	8.946625
H	19.807991	-5.588265	16.143557
H	27.070865	6.618954	5.095805
H	28.480401	5.507416	11.488545
H	31.269887	1.758561	6.877226
H	18.180496	4.922866	5.527314
H	15.796099	0.579307	10.102238
H	21.186405	-5.233914	13.991080
H	26.690397	6.042916	9.300885
H	30.295520	2.413397	9.679804
H	28.042822	3.826782	5.049776
H	16.430596	1.493071	7.163839
H	18.102118	3.550781	11.957468
H	25.021477	-5.769989	12.848208
H	23.222599	4.916504	5.983698
H	25.906637	1.897161	11.885926
H	27.914296	-0.955269	6.274101
H	20.885071	1.457953	5.071803
H	19.865194	-0.967853	11.025255
H	21.847021	-3.473605	9.838893
H	25.559943	4.928057	5.309789
H	26.484280	4.004239	10.813392
H	29.367497	0.557276	7.512698
H	20.028615	3.528768	6.021613
H	18.077936	-0.060488	9.629867
H	21.525831	-3.350674	12.267524
H	19.668662	7.396001	9.457006
H	26.670726	-1.255846	15.355653
H	25.949138	-1.989068	1.255334
H	21.104411	-2.431188	2.317457
H	21.338386	-2.438854	15.366485
H	22.668303	-6.188294	5.703523
H	19.743231	3.105093	9.081185
H	26.787205	-3.579648	11.758638
H	23.428432	-2.225864	4.733588
H	20.568373	-4.114422	6.231465
H	24.297032	0.390368	14.102894
H	26.012613	-3.532307	6.132525
H	22.057015	3.206963	8.304155
H	27.535896	-1.612771	10.512069
H	24.913087	-0.658213	5.916917
H	19.584661	-2.024179	7.002891
H	22.611454	1.532352	12.731980
H	25.629412	-3.457505	8.534890
H	18.671181	6.476271	11.516748
H	27.739028	-4.808861	13.734326
H	23.479482	-1.721820	0.539636

H	19.920273	-5.752340	4.524404
H	24.220111	-0.092855	16.703541
H	26.649912	-5.178272	4.640810
H	16.821746	6.898144	13.272343
H	27.916454	-7.258945	14.501032
H	21.941001	-2.453573	-1.412815
H	20.007458	-8.324923	4.369087
H	25.851256	-0.173619	18.702027
H	28.214956	-5.609666	2.672172
H	14.519826	6.441044	14.087061
H	26.953674	-9.168594	15.764298
H	20.467285	-4.177004	-2.428330
H	21.273856	-10.373754	3.758866
H	27.430336	-1.475562	20.109933
H	28.500347	-6.109314	0.256332
H	13.002500	4.967482	12.779156
H	24.827504	-8.886401	17.023195
H	20.097919	-6.344715	-1.267237
H	23.513269	-10.185760	2.692323
H	27.830473	-3.884018	19.636372
H	26.518203	-6.185048	-1.244076
H	13.795488	3.968133	10.651430
H	23.680864	-6.687287	17.017166
H	21.215754	-6.780112	0.904249
H	24.465955	-7.937248	2.226121
H	26.637635	-4.979640	17.755014
H	24.259974	-5.740478	-0.315771
H	20.758275	-0.425930	8.608960
H	21.504512	0.808679	7.839229
H	23.198517	1.108281	5.166831
H	22.885211	2.425802	6.083573
H	22.436457	-1.069344	6.641242
H	23.612864	-1.644888	7.600931

Table S2. Optimized Coordinates for Furfural+Fe_4 cage

C	25.766242	-12.689030	6.584066
C	26.069608	-14.027515	6.606795
C	26.515861	-14.336449	5.335320
O	26.509615	-13.253025	4.504834
C	26.041848	-12.248642	5.302623
C	26.950462	-15.675627	4.913261
H	25.384695	-12.093142	7.410392
H	25.976360	-14.703527	7.454643
H	25.905369	-11.224833	4.960937
O	27.344172	-15.871966	3.731572
H	26.934364	-16.503999	5.619074
Fe	29.548918	5.153947	7.808217
Fe	16.749220	3.974397	9.156576
Fe	23.330022	-4.847207	2.577506
Fe	24.505599	-3.828559	15.419106
S	24.214256	6.057369	10.573064
S	30.401813	0.145426	11.381948
S	26.712082	2.448930	3.084197
S	17.160005	-1.419089	6.575953
S	20.824572	3.213660	13.218232
S	26.155249	-5.989781	10.019739
S	22.184740	7.767312	7.558209
S	27.163760	1.435565	14.531451
S	28.421040	-1.713444	3.901180
S	19.527113	-0.030542	2.726936
S	19.285184	0.057069	15.320490
S	22.088199	-6.479588	8.341321
O	23.006386	7.255352	10.873171
O	30.519689	-1.302590	10.447398
O	23.770678	4.819414	11.386211
O	25.442026	6.516401	11.395285
O	26.996609	1.386615	1.996205
O	30.418617	-0.307110	12.861306
O	31.791609	0.815086	11.262069
O	15.651628	-1.150099	6.792653
O	25.050416	2.881322	2.893346
O	27.505480	3.658011	2.533541
O	20.824970	4.582688	12.497094
O	17.585621	-2.581839	7.780568
O	26.795948	-6.800254	11.171600
O	20.465426	3.578197	14.867865
O	17.130861	-2.193561	5.236911
O	22.334057	2.876679	13.187276
O	25.707298	-7.124602	9.068863
O	27.481635	-5.213876	9.230347
O	23.436243	8.338782	8.263911
O	27.241694	1.314982	16.253503
O	21.101382	9.103827	7.398401
O	25.973556	2.381309	14.246070
O	29.284443	-0.450751	3.669758
O	22.708244	7.523420	6.123147
O	28.378463	2.310996	14.141664
O	28.909952	-2.194411	5.287537
O	20.447905	1.402653	2.439718
O	19.120916	-0.161689	17.026572
O	29.031337	-2.878628	2.780602
O	19.312913	-0.656882	1.328307
O	18.093921	0.449741	3.057114
O	22.537201	-7.361757	9.757286

O	18.634647	1.427432	15.024264
O	18.260958	-0.923364	14.702326
O	21.493521	-7.540483	7.384575
O	20.822340	-5.680210	8.730128
N	30.354972	5.738389	6.001044
N	29.487414	7.081322	8.537671
N	31.411683	4.791145	8.617707
N	16.533846	5.976159	8.585840
N	14.949504	3.185972	8.546349
N	22.811071	-4.172226	16.542514
N	27.726475	5.501614	6.796694
N	28.700822	4.707660	9.663401
N	29.706402	3.151821	7.230603
N	17.600677	3.676182	7.292056
N	17.037917	2.029021	9.893815
N	23.166426	-4.120834	13.811738
N	18.334562	5.010774	9.701069
N	26.227239	-3.618360	14.241084
N	23.655816	-2.803686	2.301419
N	21.673861	-4.590956	3.829786
N	24.063279	-1.805763	15.582263
N	24.714456	-5.028301	4.137115
N	16.026065	4.538232	10.968266
N	25.047335	-5.815162	15.375399
N	22.106400	-4.603570	0.935599
N	22.845896	-6.840921	2.787633
N	25.645514	-3.444718	17.096930
N	24.985706	-5.258207	1.417807
C	31.680409	5.807652	5.654311
C	29.855272	8.238033	7.900671
C	32.207192	5.656708	9.323204
C	15.357586	6.690842	8.775762
C	14.001864	3.785727	7.759437
C	22.706910	-4.235525	17.908658
C	32.033052	6.270412	4.384291
C	29.772450	9.451859	8.587314
C	33.470745	5.235007	9.744113
C	15.047052	7.783427	7.963576
C	12.828068	3.092377	7.454163
C	21.450814	-4.417374	18.491657
C	31.030265	6.660684	3.490356
C	29.322447	9.465372	9.912699
C	33.905366	3.941594	9.432505
C	15.829276	8.043523	6.843004
C	12.646984	1.794724	7.946294
C	20.321824	-4.532298	17.673371
C	29.685654	6.590019	3.867041
C	28.957833	8.272610	10.545956
C	33.080818	3.077100	8.705214
C	16.826306	7.142468	6.482247
C	13.640568	1.192510	8.725227
C	20.452005	-4.474163	16.282346
C	29.361560	6.125656	5.145080
C	29.049941	7.072267	9.836555
C	31.822779	3.525495	8.293788
C	17.086520	6.041525	7.308979
C	14.804146	1.911434	9.013894
C	21.721637	-4.292923	15.725697
C	27.963038	5.992097	5.604238
C	28.651548	5.770031	10.423414
C	30.866179	2.654736	7.573578

C	17.719631	4.834585	6.699419
C	15.946581	1.316850	9.736683
C	21.944500	-4.253648	14.265842
C	26.441207	5.289063	7.213521
C	28.369788	3.482888	10.151705
C	28.750885	2.386611	6.636498
C	18.183465	2.537664	6.813985
C	18.067037	1.575324	10.668535
C	23.426508	-4.153989	12.468380
C	26.004762	5.744396	8.462085
C	29.362786	2.530108	10.411231
C	28.220543	2.756964	5.396517
C	17.523640	1.309806	6.913385
C	18.766702	2.449009	11.516383
C	24.496659	-4.900798	11.966731
C	24.674380	5.591754	8.872696
C	29.056533	1.316726	11.039505
C	27.313634	1.932957	4.719728
C	18.109084	0.125832	6.452702
C	19.837185	1.994166	12.299894
C	24.755965	-4.982069	10.594088
C	23.729802	5.050338	7.979568
C	27.730425	1.057166	11.440936
C	26.935641	0.699789	5.287653
C	19.388767	0.165626	5.863340
C	20.145726	0.618072	12.324161
C	23.905560	-4.325260	9.686346
C	24.148008	4.599328	6.723311
C	26.736774	2.012359	11.193092
C	27.463597	0.321918	6.529885
C	20.048754	1.394740	5.735415
C	19.449318	-0.259062	11.486450
C	22.810851	-3.599499	10.169724
C	25.485255	4.720378	6.342402
C	27.059973	3.223102	10.583164
C	28.379198	1.145583	7.187613
C	19.446114	2.570658	6.183842
C	18.434074	0.212393	10.658421
C	22.561607	-3.530293	11.541294
C	22.294183	5.043808	8.311188
C	27.378003	-0.159272	12.196855
C	26.082838	-0.249211	4.546252
C	20.019804	-1.051748	5.316576
C	21.180803	0.062671	13.215042
C	24.079784	-4.485738	8.231711
C	21.546334	6.235929	8.304508
C	27.074734	-0.104357	13.573812
C	26.640569	-1.358980	3.878096
C	20.135620	-1.252963	3.925879
C	20.933883	-0.157308	14.587321
C	23.327504	-5.437644	7.516523
C	20.237770	6.233048	8.794953
C	26.725417	-1.275077	14.251567
C	25.813250	-2.208007	3.138115
C	20.704193	-2.437149	3.444469
C	21.938692	-0.713161	15.387623
C	23.552568	-5.608314	6.146522
C	19.611473	5.031340	9.201663
C	26.649290	-2.501871	13.586935
C	24.434409	-1.989932	3.061664
C	21.177134	-3.422338	4.317175

C	23.145223	-1.159129	14.820217
C	24.493118	-4.830404	5.464729
C	20.328973	3.833820	9.056111
C	27.030754	-2.560381	12.232157
C	23.894793	-0.846898	3.677047
C	20.996179	-3.240661	5.699363
C	23.356122	-0.991728	13.447217
C	25.286328	-3.930881	6.201441
C	21.667400	3.841881	8.656677
C	27.367132	-1.396572	11.539225
C	24.707509	0.001052	4.434070
C	20.452259	-2.054917	6.195064
C	22.399669	-0.350413	12.658863
C	25.065159	-3.743318	7.567649
C	17.485258	6.005821	9.775788
C	26.877410	-4.751782	14.161754
C	23.023391	-2.360467	1.247851
C	21.101512	-5.736051	4.092914
C	24.489147	-1.327131	16.721734
C	25.896201	-5.351233	3.681466
C	16.694780	5.719902	11.021213
C	26.269490	-5.957062	14.773670
C	22.193922	-3.315755	0.474493
C	21.703176	-6.969072	3.532721
C	25.401511	-2.167982	17.531783
C	26.068467	-5.509213	2.218707
C	16.585091	6.523136	12.157790
C	26.852357	-7.223430	14.677778
C	21.465625	-2.932423	-0.654859
C	21.175649	-8.238872	3.781773
C	26.025664	-1.697230	18.689711
C	27.295025	-5.849205	1.641733
C	15.785078	6.067493	13.219006
C	26.175295	-8.324430	15.213163
C	20.666685	-3.881017	-1.301952
C	21.824009	-9.359470	3.252464
C	26.889974	-2.546685	19.388221
C	27.389284	-5.937269	0.249010
C	15.117153	4.830986	13.134950
C	24.929491	-8.155972	15.829111
C	20.598605	-5.193374	-0.819876
C	22.985479	-9.206222	2.486671
C	27.120119	-3.849734	18.931886
C	26.269225	-5.684040	-0.551149
C	15.252573	4.050760	11.980896
C	24.367995	-6.879196	15.908104
C	21.333969	-5.549565	0.313192
C	23.493591	-7.925637	2.255351
C	26.480340	-4.296667	17.773241
C	25.055673	-5.343371	0.051275
H	32.454068	5.515976	6.353011
H	30.212839	8.218717	6.878616
H	31.870593	6.660964	9.548579
H	14.696581	6.471400	9.608438
H	14.162151	4.779334	7.361681
H	23.584065	-4.144798	18.537102
H	33.077290	6.329733	4.094256
H	30.057433	10.377467	8.097293
H	34.112017	5.906962	10.305632
H	14.187388	8.406976	8.188263
H	12.066235	3.553595	6.833892

H	21.351333	-4.465966	19.571311
H	31.296785	7.019151	2.501151
H	29.254632	10.405444	10.450988
H	34.885184	3.607161	9.758446
H	15.591314	8.881303	6.195284
H	11.740617	1.247345	7.707457
H	19.342215	-4.671636	18.119433
H	28.908460	6.888424	3.169742
H	28.603206	8.286510	11.572310
H	33.415816	2.070524	8.473378
H	17.309794	7.240011	5.514367
H	13.513133	0.173829	9.079305
H	19.574886	-4.571667	15.649394
H	27.153414	6.285034	4.933435
H	28.296327	5.716348	11.454189
H	31.130804	1.619602	7.351140
H	18.176102	4.917189	5.711983
H	15.839151	0.316691	10.157004
H	21.098388	-4.421338	13.598193
H	26.660298	6.316738	9.083323
H	30.395808	2.769329	10.229845
H	28.601363	3.628097	4.893068
H	16.503075	1.281758	7.241191
H	18.475063	3.484678	11.593177
H	25.055357	-5.532793	12.621668
H	23.429487	4.182153	6.022924
H	25.712397	1.833726	11.508904
H	27.192005	-0.632223	6.973510
H	21.033662	1.442909	5.278787
H	19.695237	-1.317487	11.473800
H	22.131374	-3.107154	9.478978
H	25.773108	4.373534	5.356001
H	26.284548	3.962195	10.456554
H	28.796689	0.815368	8.133467
H	19.995898	3.501707	6.084144
H	17.949293	-0.492877	9.990384
H	21.698613	-2.965992	11.878848
H	19.707552	7.175523	8.787709
H	26.598071	-1.234960	15.318769
H	26.268082	-2.985204	2.546613
H	20.679010	-2.627516	2.386477
H	21.740746	-0.897045	16.439945
H	23.058018	-6.416494	5.637458
H	19.865586	2.880500	9.248525
H	27.016414	-3.501201	11.693266
H	22.840149	-0.617415	3.583724
H	21.286459	-4.013329	6.398762
H	24.266617	-1.342252	12.979584
H	26.068485	-3.360226	5.715538
H	22.216503	2.905289	8.614228
H	27.612398	-1.458467	10.482453
H	24.267056	0.868457	4.918175
H	20.348427	-1.926054	7.268983
H	22.591376	-0.216384	11.597645
H	25.676790	-3.030544	8.114464
H	17.921034	6.994435	9.897254
H	27.816849	-4.834682	13.612849
H	23.094115	-1.316008	0.939209
H	20.206538	-5.795853	4.714869
H	24.247159	-0.311096	17.037566
H	26.744524	-5.499138	4.352000

H	17.103639	7.474282	12.223366
H	27.810549	-7.358929	14.185078
H	21.509176	-1.912499	-1.025035
H	20.279277	-8.360733	4.382526
H	25.849359	-0.686238	19.044696
H	28.167205	-6.038742	2.260315
H	15.684902	6.672722	14.114220
H	26.614980	-9.314379	15.145557
H	20.095648	-3.597754	-2.180478
H	21.425625	-10.351978	3.437716
H	27.382095	-2.194727	20.289303
H	28.335101	-6.202028	-0.213048
H	14.509094	4.480359	13.962769
H	24.404903	-9.010526	16.244627
H	19.981230	-5.929937	-1.324313
H	23.486606	-10.075810	2.073487
H	27.784876	-4.511218	19.478395
H	26.341766	-5.755747	-1.631760
H	14.769203	3.083895	11.894596
H	23.407908	-6.737355	16.389568
H	21.294267	-6.565620	0.686025
H	24.386719	-7.799336	1.656061
H	26.638806	-5.310319	17.428005
H	24.182137	-5.157983	-0.561330

Table S3. Optimized Coordinates for Furfuralium@Fe_4

O	26.018845	1.887731	8.108643
C	24.931166	1.905073	8.743157
C	24.470010	0.704712	9.431133
O	25.206881	-0.434326	9.509848
C	23.265386	0.599590	10.083540
C	24.412304	-1.262507	10.238473
C	23.222751	-0.670396	10.581396
H	24.338082	2.811428	8.785608
H	24.697421	-2.266266	10.515937
H	22.415447	-1.123606	11.141624
H	22.501928	1.364398	10.182938
H	26.343986	2.716200	7.637683
Fe	29.537622	5.156368	7.740710
Fe	16.663426	4.249305	9.056319
Fe	23.273327	-4.932412	2.690294
Fe	24.453681	-3.842579	15.610057
S	24.238163	5.814975	10.760896
S	30.259066	-0.230882	10.723023
S	26.011842	2.676044	3.408537
S	16.811730	-1.160297	6.263467
S	20.123821	3.113607	13.917169
S	25.975056	-6.245011	10.197002
S	22.184012	8.163430	8.239262
S	27.526562	1.315215	14.563711
S	28.230247	-0.512362	2.220348
S	19.986889	0.182185	2.529598
S	18.967888	-1.668049	14.055160
S	21.621740	-6.481142	8.326298
O	22.956653	6.854798	11.271292
O	30.107373	-1.582600	9.657936
O	23.938058	4.450202	11.424988
O	25.463021	6.283047	11.582576
O	25.930889	1.853968	2.102228
O	30.459949	-0.828695	12.135848
O	31.670104	0.340658	10.445885
O	15.325365	-0.730394	6.278685
O	24.377396	3.058049	3.810597
O	26.591765	4.005001	2.867959
O	19.124259	4.241858	14.267258
O	16.949875	-2.338869	7.519076
O	26.523854	-7.222299	11.263867
O	20.467915	2.351999	15.429714
O	16.869573	-1.951478	4.935009
O	21.389890	3.925799	13.556677
O	25.556702	-7.232982	9.082316
O	27.363969	-5.416196	9.589984
O	23.496728	8.509362	8.979825
O	27.865316	1.115022	16.246248
O	21.169206	9.547324	8.439808
O	26.382319	2.354149	14.501921
O	28.625338	0.982515	2.197250
O	22.626796	8.219840	6.758197
O	28.730167	2.119733	14.017569
O	29.371373	-1.131215	3.061332
O	21.137174	1.463846	2.390400
O	18.546655	-2.601910	15.446590
O	28.517585	-1.082531	0.614794
O	19.871767	-0.408354	1.103931
O	18.606606	0.866967	2.670832

O	21.903523	-7.392789	9.766821
O	17.897254	-0.552529	13.995847
O	18.588151	-2.552247	12.844219
O	21.102887	-7.517721	7.301310
O	20.344917	-5.651628	8.598139
N	29.493225	7.040617	8.579065
N	31.433025	4.783076	8.469297
N	16.309516	6.058045	8.131697
N	14.938845	3.391915	8.331482
N	22.844578	-4.245487	16.837125
N	27.664069	5.597380	6.822056
N	28.773518	4.594385	9.615452
N	29.728278	3.178694	7.005462
N	17.669764	3.833060	7.243448
N	16.935643	2.343053	9.910021
N	23.115336	-4.458678	14.109590
N	18.318257	5.163176	9.917498
N	26.109167	-3.622856	14.327868
N	23.679134	-2.950572	2.218751
N	21.510130	-4.588879	3.778790
N	23.880130	-1.818910	15.552376
N	24.572850	-4.939258	4.358311
N	15.744018	4.872392	10.796505
N	25.160685	-5.777583	15.748292
N	22.171504	-4.847048	0.945543
N	22.781442	-6.903218	3.025727
N	25.620266	-3.191495	17.181711
N	25.003787	-5.399725	1.673305
C	31.598202	5.928981	5.555391
C	29.823214	8.234930	7.991196
C	32.218654	5.607279	9.234952
C	15.550121	7.104059	8.591329
C	14.013728	3.953786	7.491099
C	22.760736	-4.081719	18.195901
C	31.910925	6.475312	4.308614
C	29.766685	9.408359	8.747087
C	33.507941	5.197079	9.582360
C	15.474568	8.278116	7.838532
C	12.866168	3.230192	7.157623
C	21.594334	-4.467671	18.860672
C	30.882727	6.946136	3.486175
C	29.383044	9.344520	10.091236
C	33.980734	3.959020	9.134919
C	16.172851	8.366824	6.629549
C	12.679943	1.949244	7.690559
C	20.536146	-5.021511	18.131400
C	29.553349	6.871713	3.911833
C	29.060646	8.114712	10.673776
C	33.168495	3.140222	8.344830
C	16.932545	7.284388	6.174764
C	13.637306	1.396316	8.547594
C	20.645450	-5.187518	16.747126
C	29.269607	6.317812	5.164356
C	29.125977	6.956321	9.894405
C	31.882660	3.576707	8.011889
C	16.989963	6.121127	6.948390
C	14.774592	2.143617	8.866299
C	21.824518	-4.795273	16.107391
C	27.890818	6.204810	5.677588
C	28.779665	5.621372	10.430432
C	30.950027	2.749126	7.220241

C	17.719577	4.915379	6.505848
C	15.875711	1.601243	9.694659
C	21.992839	-4.875471	14.638199
C	26.364589	5.444375	7.251945
C	28.402371	3.362233	10.071797
C	28.815461	2.358118	6.394715
C	18.193617	2.655500	6.780298
C	17.977499	1.866004	10.656293
C	23.306137	-4.489228	12.759267
C	25.984723	5.689939	8.580417
C	29.289884	2.278193	10.042249
C	27.980636	2.822760	5.368476
C	17.449153	1.472387	6.825270
C	18.530410	2.621922	11.698631
C	24.386895	-5.186244	12.209736
C	24.641789	5.620296	8.991704
C	28.994476	1.073782	10.702907
C	27.114531	1.958327	4.675541
C	17.919480	0.281841	6.255037
C	19.536722	2.101317	12.528569
C	24.563503	-5.287630	10.824717
C	23.635869	5.358987	8.039926
C	27.774401	0.941887	11.403860
C	27.111625	0.579179	4.989359
C	19.185719	0.256297	5.628051
C	20.009378	0.788025	12.316670
C	23.626974	-4.691627	9.955552
C	23.995581	5.111061	6.713983
C	26.871811	2.011604	11.400602
C	27.925842	0.110667	6.025412
C	19.921641	1.447109	5.550901
C	19.475585	0.038675	11.258374
C	22.507000	-4.043299	10.498135
C	25.334837	5.145909	6.327621
C	27.196949	3.211095	10.775923
C	28.766668	0.982218	6.714828
C	19.429554	2.631046	6.100706
C	18.457926	0.557221	10.457377
C	22.339650	-3.956379	11.883528
C	22.208207	5.341119	8.414596
C	27.432637	-0.261554	12.199495
C	26.271578	-0.394004	4.260962
C	19.781872	-1.000500	5.101822
C	20.994691	0.140819	13.220698
C	23.815474	-4.718928	8.487458
C	21.502627	6.534289	8.669340
C	27.180996	-0.179244	13.587788
C	26.605346	-0.842568	2.965924
C	20.239230	-1.124393	3.768119
C	20.663492	-1.021437	13.951939
C	22.971066	-5.470792	7.648581
C	20.208956	6.469041	9.199248
C	26.724879	-1.305975	14.277973
C	25.709314	-1.663876	2.270178
C	20.847016	-2.317300	3.348317
C	21.645903	-1.674218	14.701696
C	23.224393	-5.517085	6.270959
C	19.561533	5.229857	9.371992
C	26.506688	-2.522608	13.627219
C	24.545842	-2.152618	2.894724
C	20.990519	-3.405797	4.215776

C	22.965684	-1.216168	14.739492
C	24.285168	-4.810619	5.692332
C	20.225500	4.060336	8.988949
C	26.867235	-2.625188	12.270095
C	24.302546	-1.821484	4.230219
C	20.490823	-3.288205	5.525787
C	23.270382	-0.009776	14.080436
C	25.131122	-4.073111	6.549864
C	21.548341	4.111473	8.547213
C	27.304002	-1.503514	11.560972
C	25.144651	-0.928940	4.899345
C	19.920590	-2.095813	5.967785
C	22.298935	0.654686	13.322469
C	24.896919	-4.027825	7.924220
C	17.964569	5.874216	10.957461
C	26.818136	-4.722856	14.300502
C	23.264811	-2.665930	1.011617
C	20.885798	-5.720039	3.999960
C	24.433195	-1.156124	16.535672
C	25.809338	-5.103850	3.954168
C	16.593583	5.694009	11.490179
C	26.335476	-5.900131	15.056317
C	22.395539	-3.647362	0.322571
C	21.542255	-6.986117	3.600155
C	25.370548	-1.868893	17.435431
C	26.072687	-5.359842	2.523397
C	16.168870	6.285050	12.683001
C	27.010947	-7.123671	15.043463
C	21.787617	-3.377765	-0.906328
C	20.983133	-8.238875	3.866533
C	26.029210	-1.227287	18.488007
C	27.360150	-5.597153	2.032713
C	14.872680	6.030694	13.142878
C	26.474913	-8.203196	15.752386
C	20.961576	-4.348245	-1.482472
C	21.703784	-9.389340	3.528895
C	26.927884	-1.957091	19.272697
C	27.528879	-5.875879	0.672819
C	14.016798	5.196546	12.414580
C	25.278295	-8.055237	16.462175
C	20.753519	-5.571643	-0.835420
C	22.968751	-9.281725	2.938924
C	27.161733	-3.310155	19.002949
C	26.420089	-5.922416	-0.179064
C	14.465953	4.619412	11.224268
C	24.622734	-6.821711	16.456560
C	21.375510	-5.816510	0.391290
C	23.504386	-8.016477	2.685618
C	26.491848	-3.926521	17.943408
C	25.145433	-5.679782	0.337982
N	30.285450	5.854266	5.949196
O	20.623883	0.542991	8.388546
O	23.435566	1.563241	6.131056
O	22.638703	-1.788011	7.353724
H	32.394575	5.580270	6.199427
H	30.133511	8.277183	6.954918
H	31.857261	6.572819	9.564202
H	15.009208	7.028769	9.526361
H	14.163388	4.948650	7.089448
H	23.588018	-3.665652	18.756983
H	32.944401	6.539071	3.983190

H	30.022649	10.361902	8.296170
H	34.139861	5.836487	10.190546
H	14.879419	9.115278	8.189303
H	12.123614	3.659101	6.492307
H	21.511205	-4.342507	19.935584
H	31.117799	7.373783	2.516608
H	29.336519	10.252488	10.684169
H	34.980906	3.632888	9.402051
H	16.118326	9.275758	6.038759
H	11.792422	1.380106	7.432776
H	19.626091	-5.320950	18.641531
H	28.756613	7.240118	3.272367
H	28.760588	8.067728	11.716411
H	33.534950	2.176417	8.003984
H	17.461645	7.350427	5.228719
H	13.499861	0.395206	8.945179
H	19.818472	-5.606357	16.181501
H	27.083561	6.621786	5.073499
H	28.482877	5.522711	11.476494
H	31.288169	1.774691	6.863721
H	18.186282	4.916908	5.519520
H	15.786521	0.594691	10.106772
H	21.182670	-5.268693	14.021483
H	26.695646	6.050391	9.283819
H	30.274967	2.404993	9.638564
H	28.070239	3.830776	5.021774
H	16.439631	1.493508	7.171876
H	18.097458	3.567486	11.957713
H	25.032887	-5.756816	12.848392
H	23.232079	4.901623	5.970073
H	25.917324	1.927198	11.912964
H	27.919771	-0.942536	6.293373
H	20.891664	1.457345	5.071691
H	19.834959	-0.967954	11.064692
H	21.772258	-3.580707	9.845093
H	25.567258	4.940890	5.290268
H	26.501385	4.032109	10.841657
H	29.378091	0.577437	7.515909
H	20.036139	3.528517	6.022426
H	18.062637	-0.059254	9.654706
H	21.467220	-3.442062	12.275111
H	19.680472	7.391382	9.422177
H	26.667980	-1.254409	15.347718
H	25.954290	-1.984835	1.261636
H	21.094199	-2.428556	2.308655
H	21.336718	-2.467081	15.354061
H	22.675892	-6.221449	5.687362
H	19.749608	3.095795	9.097742
H	26.767488	-3.568595	11.744535
H	23.429785	-2.211053	4.738266
H	20.579841	-4.113191	6.225256
H	24.271963	0.404894	14.132725
H	25.972265	-3.512292	6.153589
H	22.062394	3.184950	8.321557
H	27.506755	-1.598404	10.498465
H	24.921486	-0.649374	5.922727
H	19.598246	-2.023339	7.001934
H	22.576260	1.556477	12.785286
H	25.569291	-3.452273	8.553162
H	18.684689	6.488436	11.499989
H	27.742099	-4.793045	13.723521

H	23.480562	-1.701657	0.548680
H	19.920640	-5.751469	4.508481
H	24.214423	-0.100294	16.706118
H	26.642228	-5.155204	4.656733
H	16.833354	6.927488	13.253030
H	27.936631	-7.242298	14.488199
H	21.946354	-2.428474	-1.409222
H	20.009503	-8.324396	4.339770
H	25.854319	-0.175746	18.695236
H	28.221214	-5.572046	2.693966
H	14.529198	6.481832	14.068419
H	26.988757	-9.159282	15.751757
H	20.481668	-4.152640	-2.436181
H	21.282897	-10.369249	3.730709
H	27.446207	-1.472112	20.093821
H	28.522581	-6.062894	0.278252
H	13.008946	5.004351	12.768703
H	24.864363	-8.891623	17.016393
H	20.118490	-6.327549	-1.286428
H	23.527924	-10.174418	2.677349
H	27.855444	-3.877782	19.615009
H	26.549427	-6.143711	-1.233749
H	13.800623	3.990047	10.647389
H	23.703994	-6.699200	17.015837
H	21.233485	-6.769148	0.885068
H	24.478693	-7.922728	2.221350
H	26.658955	-4.976439	17.738200
H	24.283349	-5.715163	-0.316340
H	20.750390	-0.450741	8.639365
H	21.478598	0.794527	7.868077
H	23.203199	1.096670	5.239313
H	22.879554	2.432905	6.121781
H	22.438237	-1.061277	6.648189
H	23.613739	-1.602908	7.629386

Table S4. Composition of the HOMO and LUMO of Furfural and Furfuralium

	Furfural				Furfuralium			
	exohedral		*endohedral*		*exohedral*		*endohedral*	
	HOMO	LUMO	HOMO	LUMO	HOMO	LUMO	HOMO	LUMO
O1	0.002	0.060	0.000	0.062	0.000	0.048	0.002	0.009
C2	0.051	0.024	0.044	0.031	0.297	0.001	0.275	0.141
C3	0.008	0.128	0.005	0.119	0.023	0.171	0.046	0.006
C4	0.002	0.000	0.001	0.000	0.162	0.002	0.097	0.015
C5	0.001	0.158	0.000	0.154	0.288	0.183	0.253	0.014
C6	0.025	0.254	0.016	0.230	0.079	0.382	0.078	0.485
O2	0.700	0.188	0.715	0.196	0.112	0.151	0.099	0.134

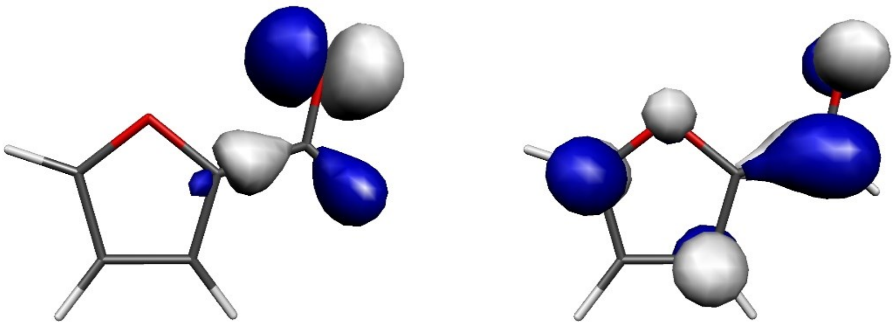

Figure S2. Isosurface plot of the HOMO (left) and LUMO (right) for furfural

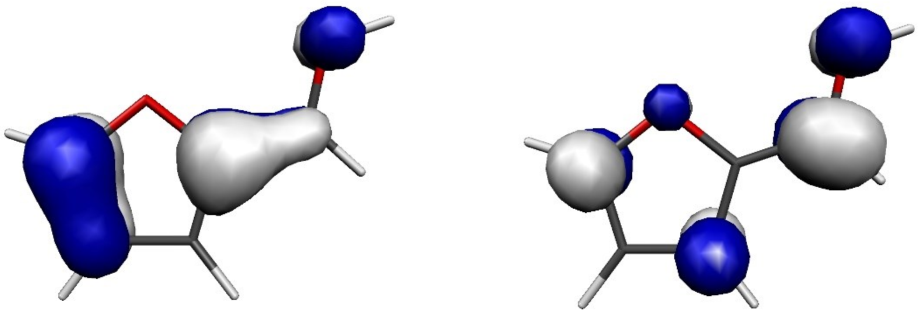

Figure S3. Isosurface plot of the HOMO (left) and LUMO (right) for the furfuralium cation

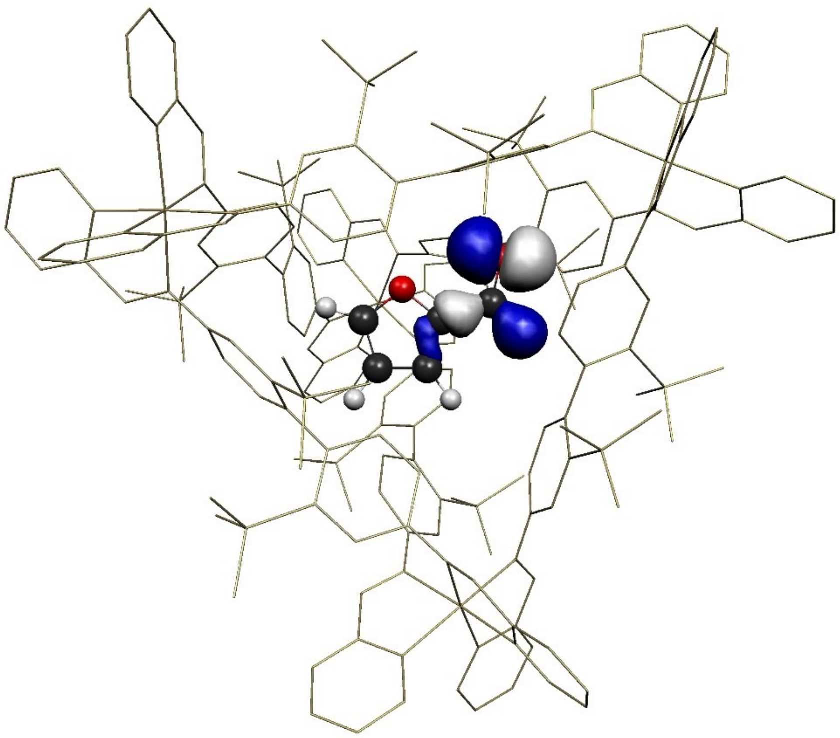

Figure S4. Isosurface plot of the HOMO of furfural@Fe_4

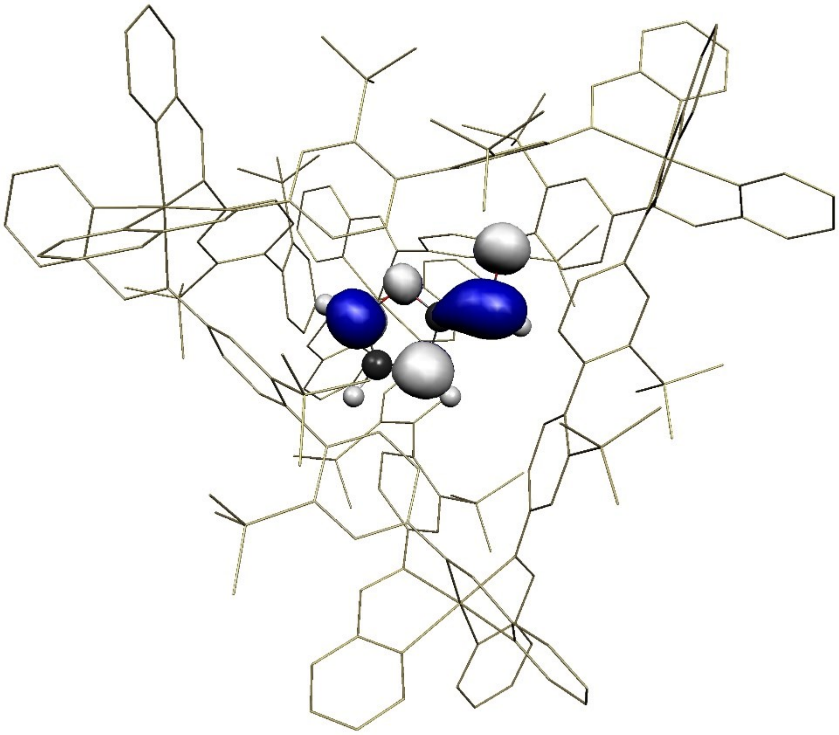

Figure S5. Isosurface plot of the LUMO of furfural@Fe_4

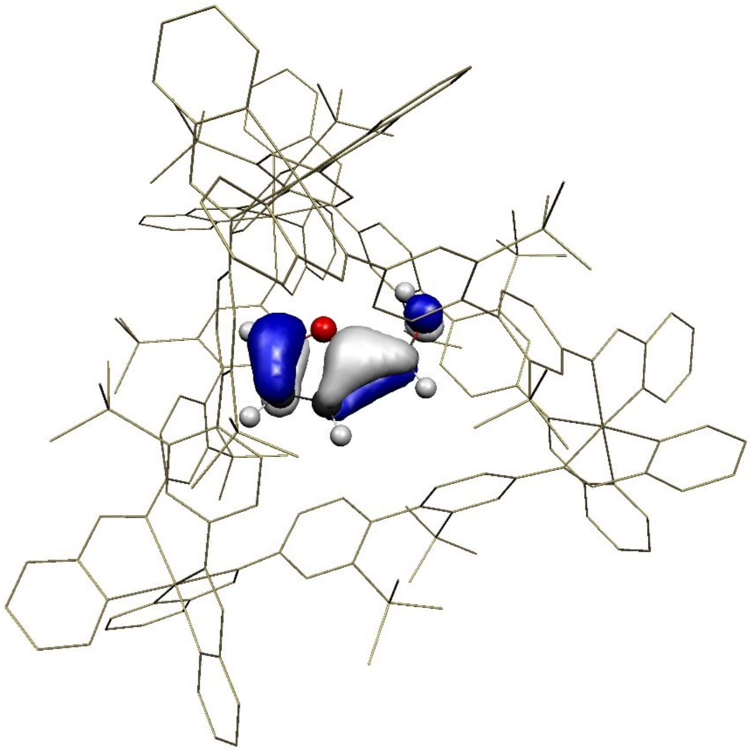

Figure S6. Isosurface plot of the HOMO of furfuralium@Fe_4

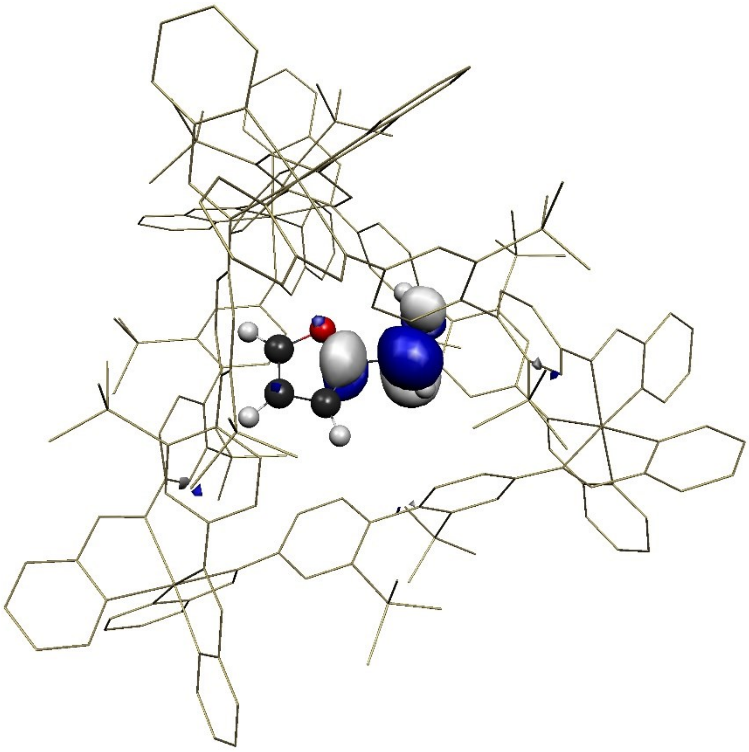

Figure S7. Isosurface plot of the LUMO of furfuralium@Fe_4

Table S5. Geometry Optimized Coordinates for Furfural

C	25.766242003	-12.689030002	6.584066001
C	26.069608003	-14.027515002	6.606795001
C	26.515861003	-14.336449002	5.335320001
O	26.509615003	-13.253025002	4.504834001
C	26.041848003	-12.248642001	5.302623001
C	26.950462003	-15.675627002	4.913261001
O	27.344172003	-15.871966002	3.731572000
H	25.384695003	-12.093142001	7.410392001
H	25.976360003	-14.703527002	7.454643001
H	25.905369003	-11.224833001	4.960937001
H	26.934364003	-16.503999002	5.619074001

Table S6. Geometry Optimized Coordinates for Furfuralium

O	23.339822	2.004865	7.568671
C	23.862178	1.038313	8.190501
C	23.102588	-0.199655	8.396733
O	21.828582	-0.379645	7.949597
C	23.586937	-1.306869	9.056753
C	21.531636	-1.645752	8.355541
C	22.580227	-2.231573	9.029226
H	24.878845	1.121652	8.575038
H	24.567060	-1.426124	9.507588
H	20.578265	-2.127409	8.165368
H	22.603226	-3.229820	9.453826
H	23.853968	2.862093	7.420399

Calculations on *p-tert*-butylbenzaldehyde

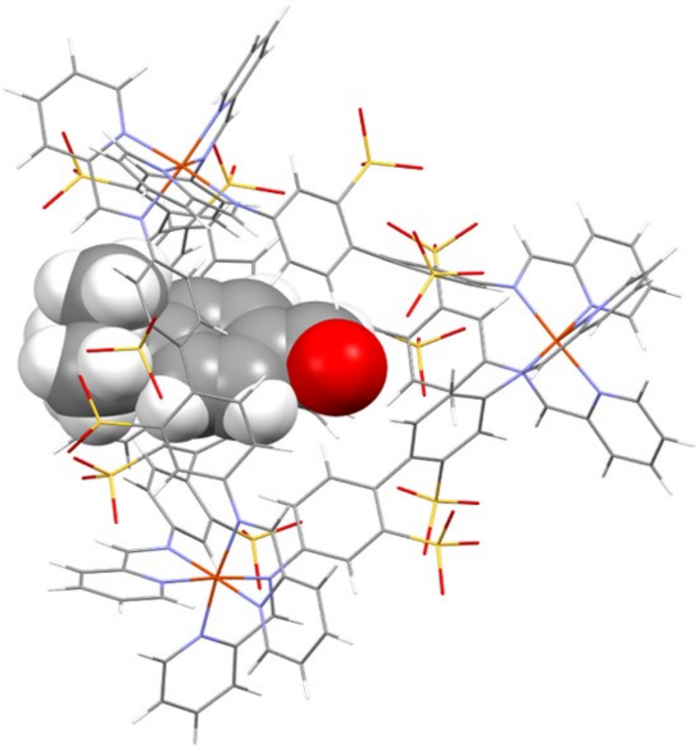

Figure S8: Energy-minimized structure for the *p-tert*-butylbenzaldehyde substrate (space-filled) encapsulated within the Fe_4L_6 cage, orientated with aldehyde functionality enclosed within the cage. This, and similar (partially) encapsulated *p-tert*-butylbenzaldehyde@Fe_4L_6 species are around 21 kcal mol^{-1} less stable than "free" *p-tert*-butylbenzaldehyde outside the cage. The length of the substrate exceeds the interior dimensions of the cage such that (in this example) the bulky t-butyl functionality protrudes through one of the triangular faces of the cage.

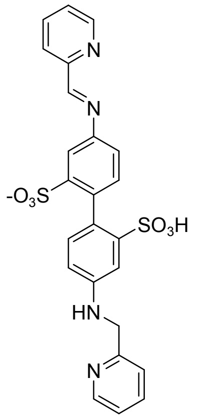

Exact Mass: 523.0751
m/z: 523.0746 (100.0%)

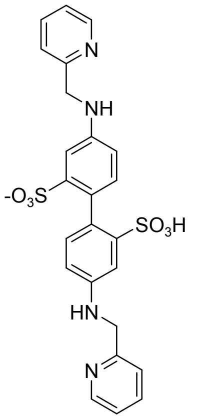

Exact Mass: 525.0908
m/z: 525.0903 (100.0%)

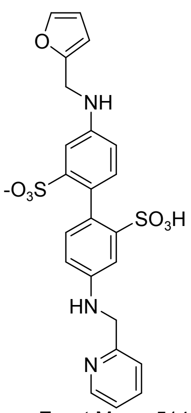

Exact Mass: 514.0748
m/z: 514.0743 (100.0%)

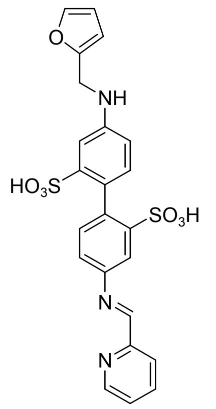

Exact Mass: 513.0664
m/e: 513.0664 (100.0%)

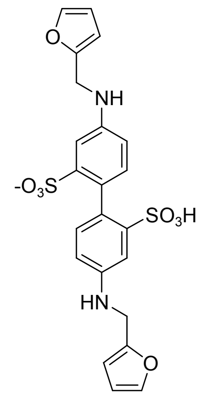

Exact Mass: 503.0588
m/z: 503.0583 (100.0%)

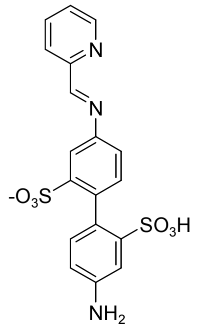

Exact Mass: 432.0330
m/e: 432.0324 (100.0%)

Figure S9: LCMS (negative mode) analysis of the aqueous (cage-containing) phase after reaction shows (in addition to intact cage) a number of minor components with the masses indicated above. The peaks at 432.0, 523.1 and 525.1 corresponding to cage ligands in various states of reduction whilst the peaks at 503.1, 513.0 and 514.1 correspond to the reduced products of scrambling reactions between furfural and the cage ligands. Hence there is a small amount of decomposition of the cage with each addition of hydride and/or aldehyde. These insights not only account for the discrepancy between furfural conversion and yield of furfuryl alcohol, but also suggest why reaction yields decrease in subsequent catalytic cycles.

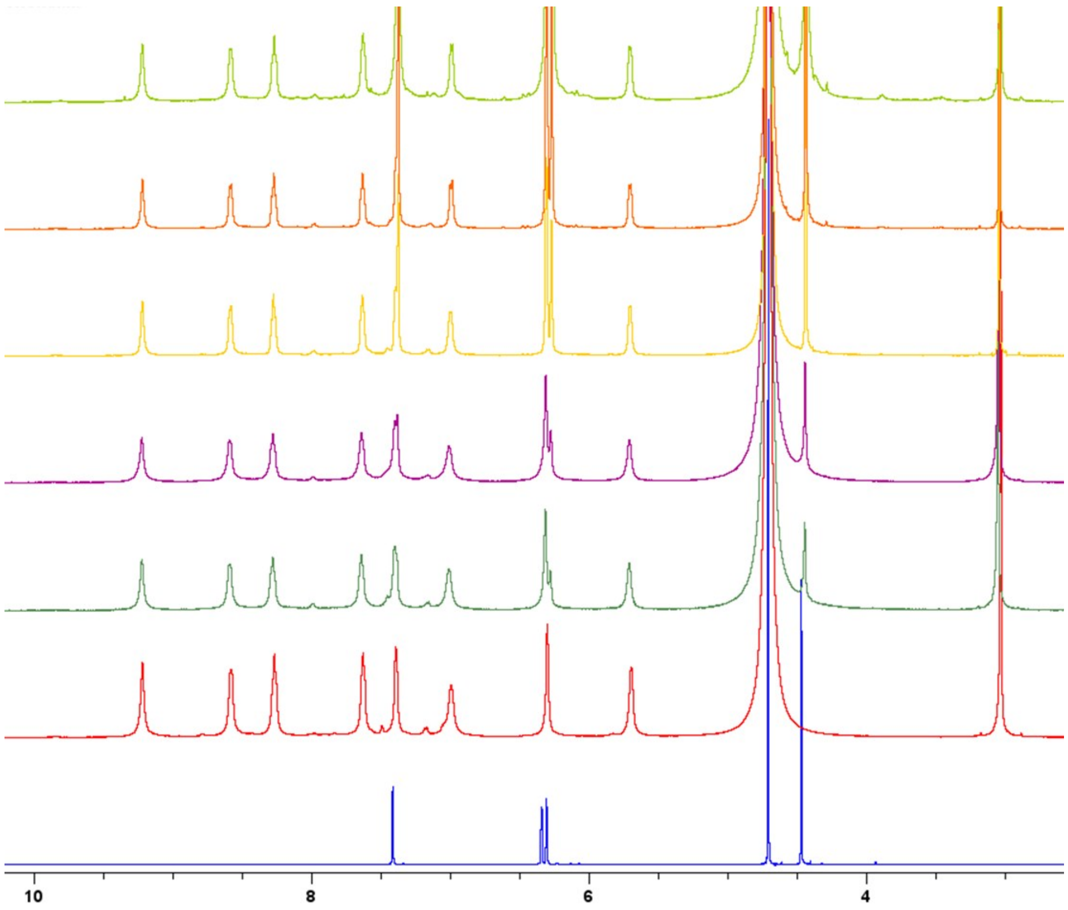

Figure S10: Stacked ^1H NMR spectra (500 MHz) in D_2O at 298 K showing free furfuryl alcohol (blue spectrum, bottom), free $Fe^{II}{}_4L_6$ cage on its own (red spectrum, second bottom) and then mixtures of cage and furfuryl alcohol (after equilibration at 50 °C for 6 h) in the following ratios (cage : furfuryl alcohol); 1:6 (dark green), 1:13 (purple), 1:25 (yellow), 1:50 (orange) and 1:100 (light green, uppermost spectrum). There are no obvious peak shifts or new peaks for encapsulated furfuryl alcohol, even at 100-fold excess of the guest (light green spectrum at the top). Meanwhile, peaks for free furfuryl alcohol (bottom spectrum) are not obviously shifted by addition of cage. Moreover, the overlap of these peaks with cage peaks makes observing any slight shifts that there may be impossible. All this suggests a very minimal association constant. The poor encapsulation of furfuryl alcohol relative to furan could be as a result of furfuryl alcohol's greater electron-richness (disfavouring association with the anionic cage), and is also probably a function of their relative water solubilities: furfural has a solubility of 83 g/L in water at room temperature, but furfuryl alcohol is fully miscible in water at this temperature.

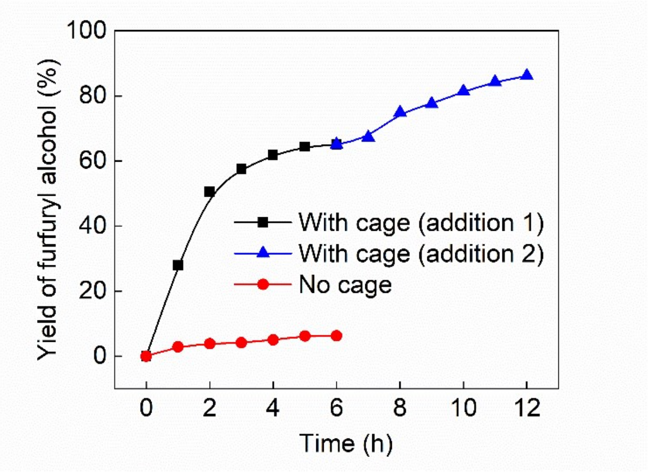

Figure S11: Yields of furfuryl alcohol *vs.* time in the presence of 9 mol% cage or in the absence of cage. Yields in the presence of cage (black line and squares for the first addition of furfural and $NaCNBH_3$, and blue line and triangles for the second addition) are isolated yields. Yields in the absence of cage (red line and circles) were determined by ^1H NMR and are likely to be slight over-estimates of the amount of furfuryl alcohol produced. The blue trace in the figure above shows the effects of adding a further equivalent of both furfural and $NaCNBH_3$ to an ongoing catalytic reaction at t = 6 h. Typically, conversion of a further 20% of this furfural was then achieved over the following 6 h, corresponding to a further two turnovers of the cage. Alternatively, after a single catalytic run, the Fe_4L_6 cage could be recovered from the aqueous phase (after extraction of the organics) by precipitation with acetone. After centrifugation and recrystallization from water/acetone, the recovered cage could then be re-used in catalytic experiments (albeit delivering conversion rates of only half that of fresh cage). Taken together, these data suggest that the cage can (at least to some extent, and notwithstanding the degradation pathways mentioned above) be recycled and re-used for more than one catalytic reaction, preforming multiple turnovers in each experiment.

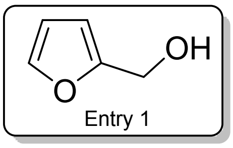

Entry 1: 100 mg (0.027 mmol) of [Fe_4L_6] cage, 29 mg (0.3 mmol) of furfural and 19 mg (0.3 mmol) of $NaCNBH_3$ were taken in 5 mL distilled water under a nitrogen atmosphere and stirred for 6 hours at 50 °C. Isolated yield: 65% (19 mg, 0.19 mmol). ^1H NMR (500 MHz, $CDCl_3$) δ = 7.39 (s, J = 1.1 Hz, 1H), 6.34 (d, J = 3.1 Hz, 1H), 6.28 (d, J = 3.2 Hz, 1H), 4.59 (s, 2H), 2.06 (br s, 1H). ^{13}C NMR (126 MHz, $CDCl_3$) δ = 154.15, 142.71, 110.50, 107.89, 57.56. Mass spectroscopy (ESI): mass calculated for $C_5H_6NaO_2$ ($[M+Na]^+$): 121.0266; Mass found: 121.0239.

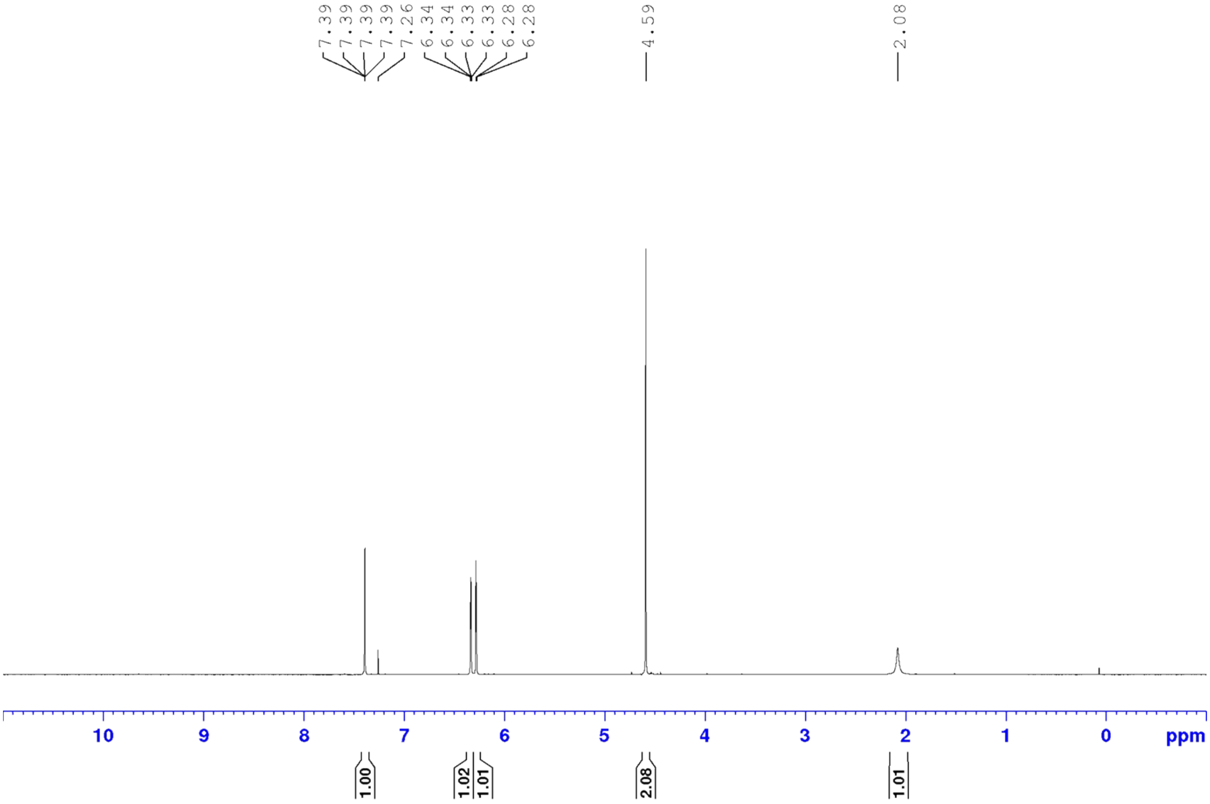

Figure S12: ^1H NMR of extracted furfuryl alcohol [$CDCl_3$, 500 MHz]

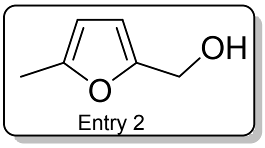

Entry 2: 100 mg (0.027 mmol) of [Fe_4L_6] cage, 33 mg (0.3 mmol) of 5-methylfurfural and 19 mg (0.3 mmol) of $NaCNBH_3$ were taken in 5 mL distilled water under a nitrogen atmosphere and stirred for 6 hours at 50 °C. Isolated yield: 51% (17 mg, 0.15 mmol). ^{1}H NMR (500 MHz, $CDCl_3$) δ = 6.17 (d, *J* = 3.0 Hz, 1H), 5.91 (m, 1H), 4.55 (d, *J* = 6.0 Hz, 2H), 2.29 (s, 3H), 1.66 (t, *J* = 6.0 Hz, 1H). ^{13}C NMR (126 MHz, $CDCl_3$) δ = 152.62, 152.38, 108.94, 106.39, 57.75, 13.72. Mass spectroscopy (ESI): mass calculated for $C_6H_8NaO_2$ ([M+Na]$^+$): 135.0422; Mass found: 135.0403.

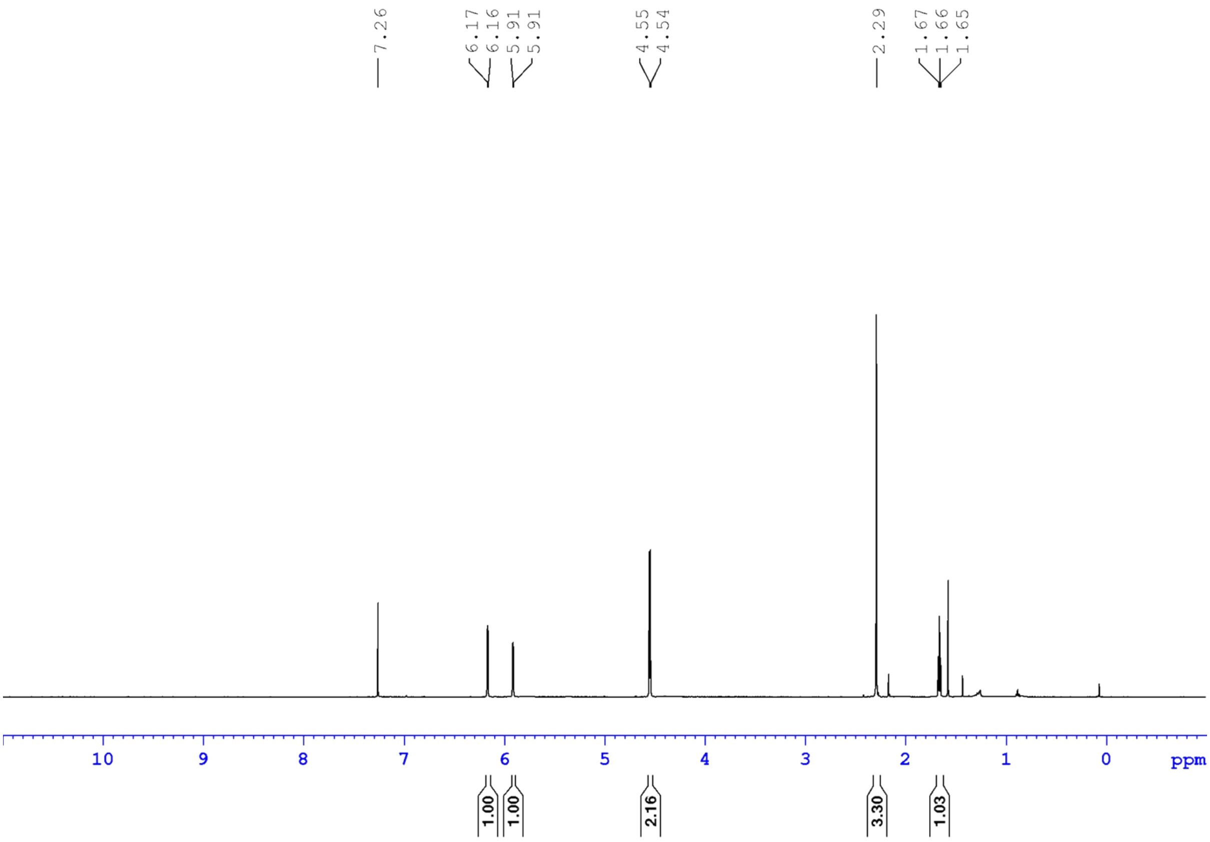

Figure S13: ^{1}H NMR of extracted 5-methylfurfuryl alcohol [$CDCl_3$, 500 MHz]

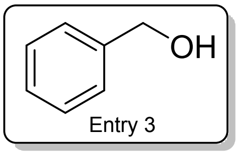

Entry 3: 100 mg (0.027 mmol) of [Fe_4L_6] cage, 32 mg (0.3 mmol) of benzaldehyde and 19 mg (0.3 mmol) of $NaCNBH_3$ were taken in 5 mL distilled water under a nitrogen atmosphere and stirred for 6 hours at 50 °C. Isolated yield: 62% (20 mg, 0.19 mmol). ^{1}H NMR (500 MHz, $CDCl_3$) δ = 7.39-7.28 (m, 5H), 4.68 (s, 2H), 1.99 (br s, 1H). ^{13}C NMR (126 MHz, $CDCl_3$) δ = 141.00, 128.70, 127.79, 127.14, 65.47. Mass spectroscopy (ESI): mass calculated for C_7H_8NaO ([M+Na]$^+$): 131.0473; Mass found: 131.1604.

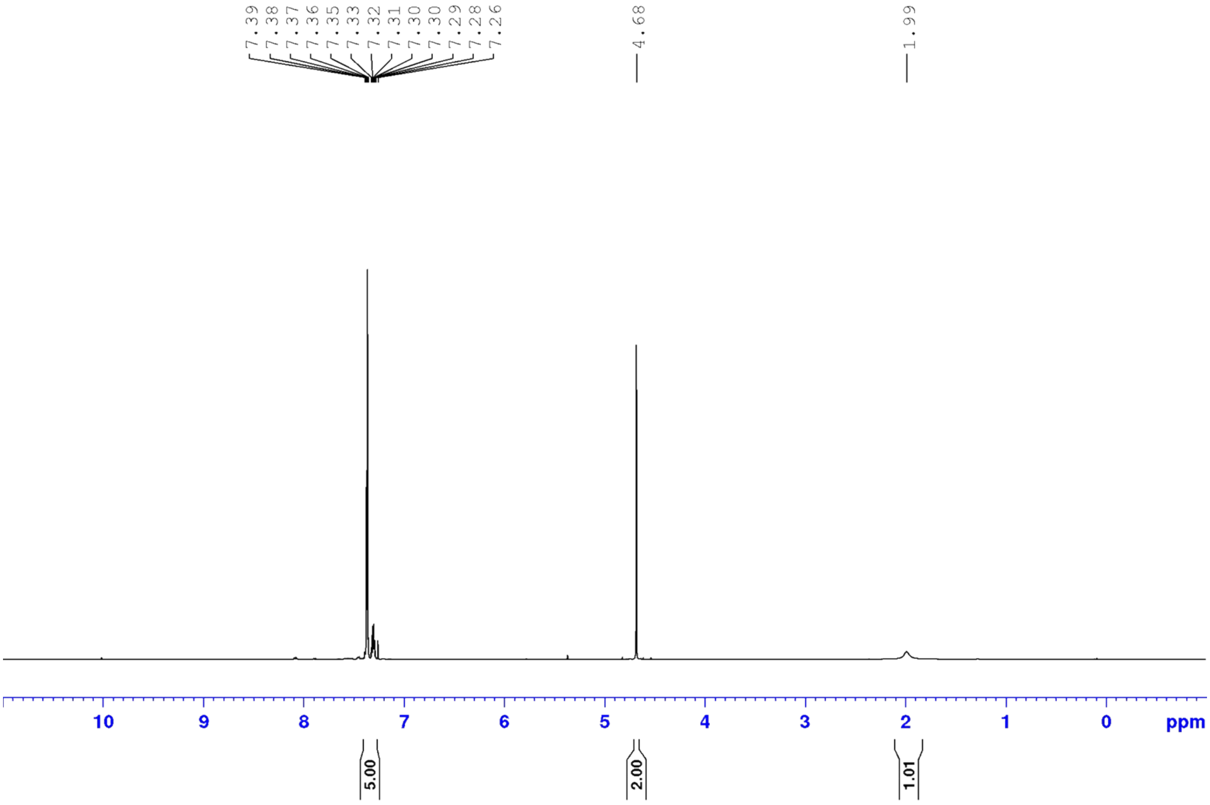

Figure S14: ^{1}H NMR of extracted benzyl alcohol [$CDCl_3$, 500 MHz]

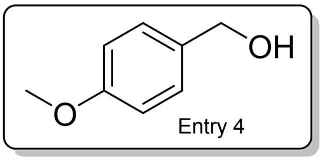

Entry 4: 100 mg (0.027 mmol) of $[Fe_4L_6]$ cage, 41 mg (0.3 mmol) of 4-methoxybenzaldehyde and 19 mg (0.3 mmol) of $NaCNBH_3$ were taken in 5 mL distilled water under a nitrogen atmosphere and stirred for 6 hours at 50 °C. Yield: 51% (21 mg, 0.15 mmol). ^1H NMR (500 MHz, $CDCl_3$) δ = 7.28 (d, *J* = 8.6 Hz, 2H), 6.89 (d, *J* = 8.6 Hz, 2H), 4.60 (s, 2H), 3.81 (s, 3H), 1.77 (br s, 1H). ^{13}C NMR (126 MHz, $CDCl_3$) δ = 159.36, 133.29, 128.80, 114.11, 65.17, 55.45. Mass spectroscopy (ESI): mass calculated for $C_8H_{10}NaO_2$ ($[M+Na]^+$): 161.0579; Mass found: 161.0569.

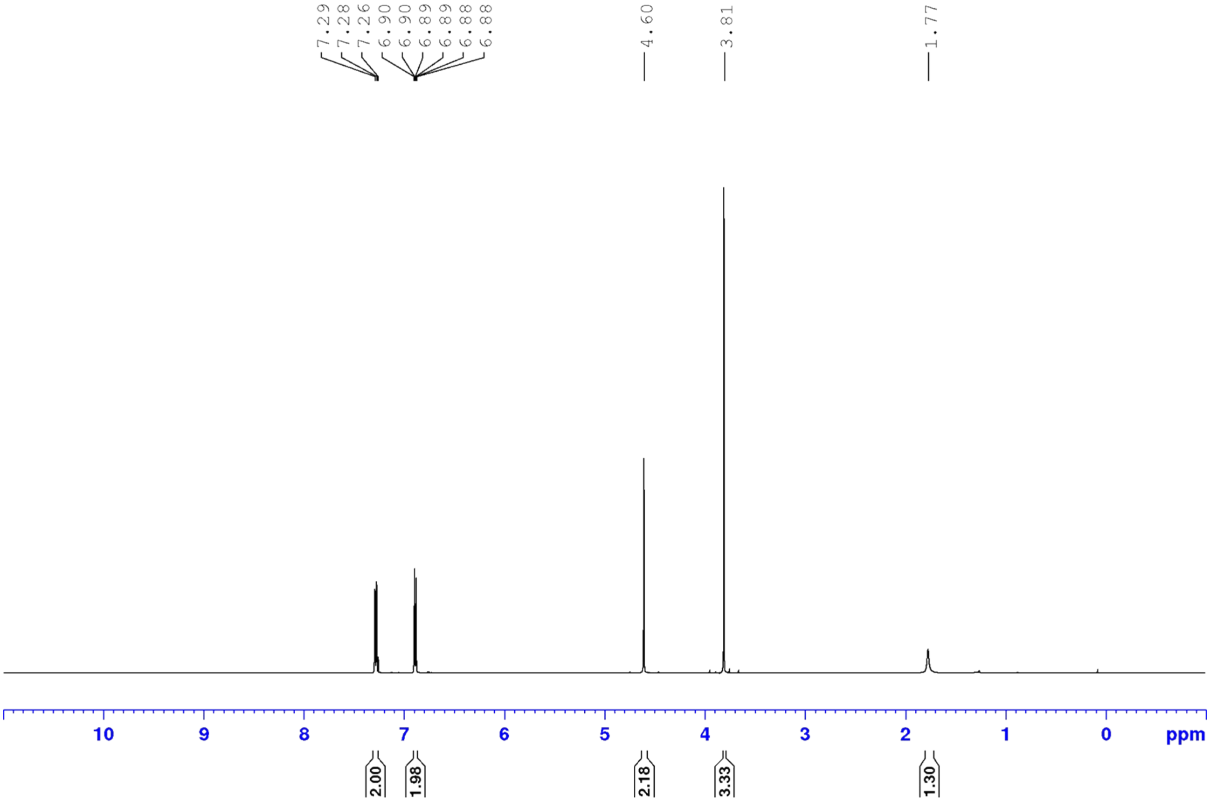

Figure S15: ^1H NMR of extracted 4-methoxybenzyl alcohol (entry 4) [$CDCl_3$, 500 MHz]

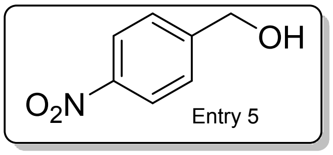

Entry 5: 100 mg (0.027 mmol) of [Fe_4L_6] cage, 45 mg (0.3 mmol) of 4-nitrobenzaldehyde and 19 mg (0.3 mmol) of $NaCNBH_3$ were taken in 5 mL distilled water under a nitrogen atmosphere and stirred for 6 hours at 50 °C. Yield: 60% (27 mg, 0.18 mmol). ^1H NMR (500 MHz, $CDCl_3$) δ = 8.20 (d, *J* = 8.6 Hz, 2H), 7.52 (d, *J* = 8.6 Hz, 2H), 4.83 (s, 2H), 2.12 (br s, 1H). ^{13}C NMR (126 MHz, $CDCl_3$) δ = 148.36, 147.44, 127.16, 123.89, 64.15. Mass spectroscopy (ESI): mass calculated for $C_7H_7NNaO_3$ ($[M+Na]^+$): 176.0324; Mass found: 176.0571.

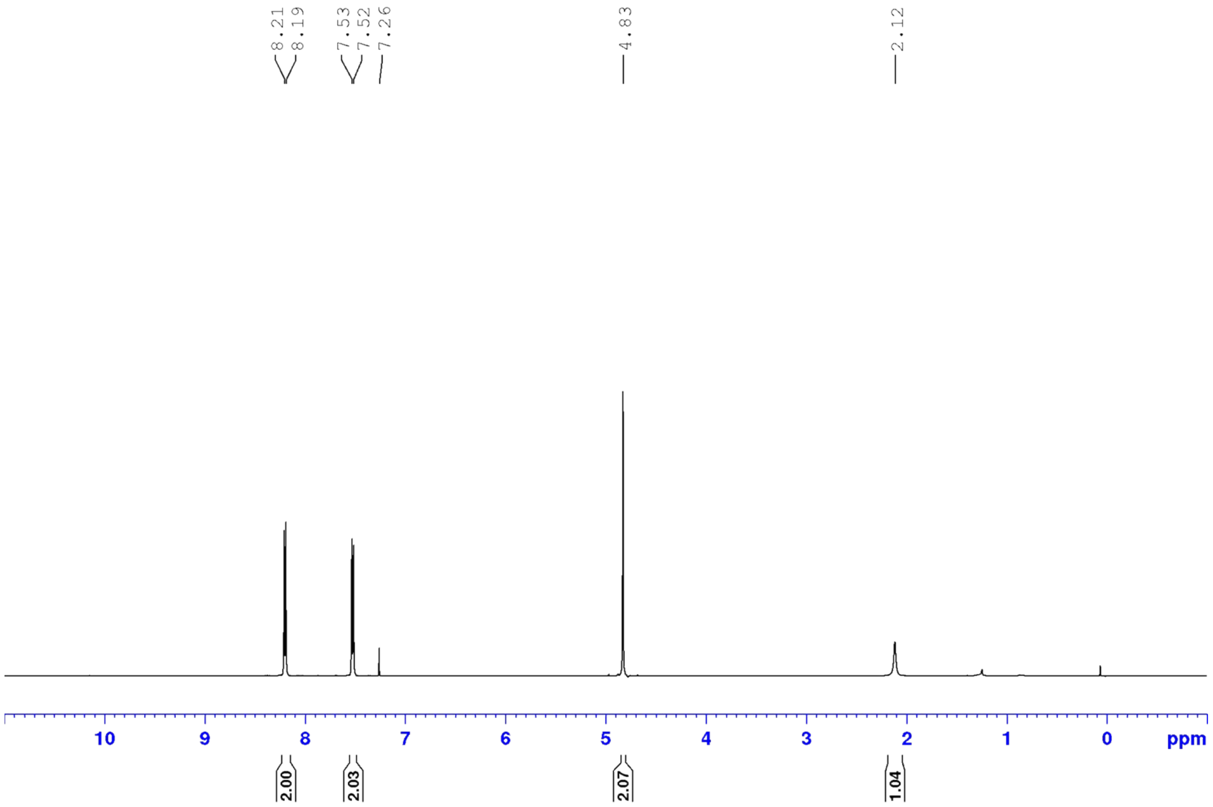

Figure S16: ^1H NMR of extracted 4-nitrobenzyl alcohol [$CDCl_3$, 500 MHz]

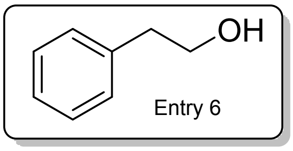

Entry 6: 100 mg (0.027 mmol) of [Fe_4L_6] cage, 36 mg (0.3 mmol) of phenylacetaldehyde and 19 mg (0.3 mmol) of $NaCNBH_3$ were taken in 5 mL distilled water under a nitrogen atmosphere and stirred for 6 hours at 50 °C. Yield: 43% (15 mg, 0.12 mmol). ^{1}H NMR (500 MHz, $CDCl_3$) δ = 7.35-7.24 (m, 5H), 3.89 (t, J = 6.6 Hz, 2H), 2.90 (t, J = 6.6 Hz, 2H), 1.43 (br s, 1H). ^{13}C NMR (126 MHz, $CDCl_3$) δ = 138.64, 129.22, 128.78, 126.67, 63.88, 39.39. Mass spectroscopy (ESI): mass calculated for $C_8H_{10}NaO$ ($[M+Na]^+$): 145.0629; Mass found: 145.0607.

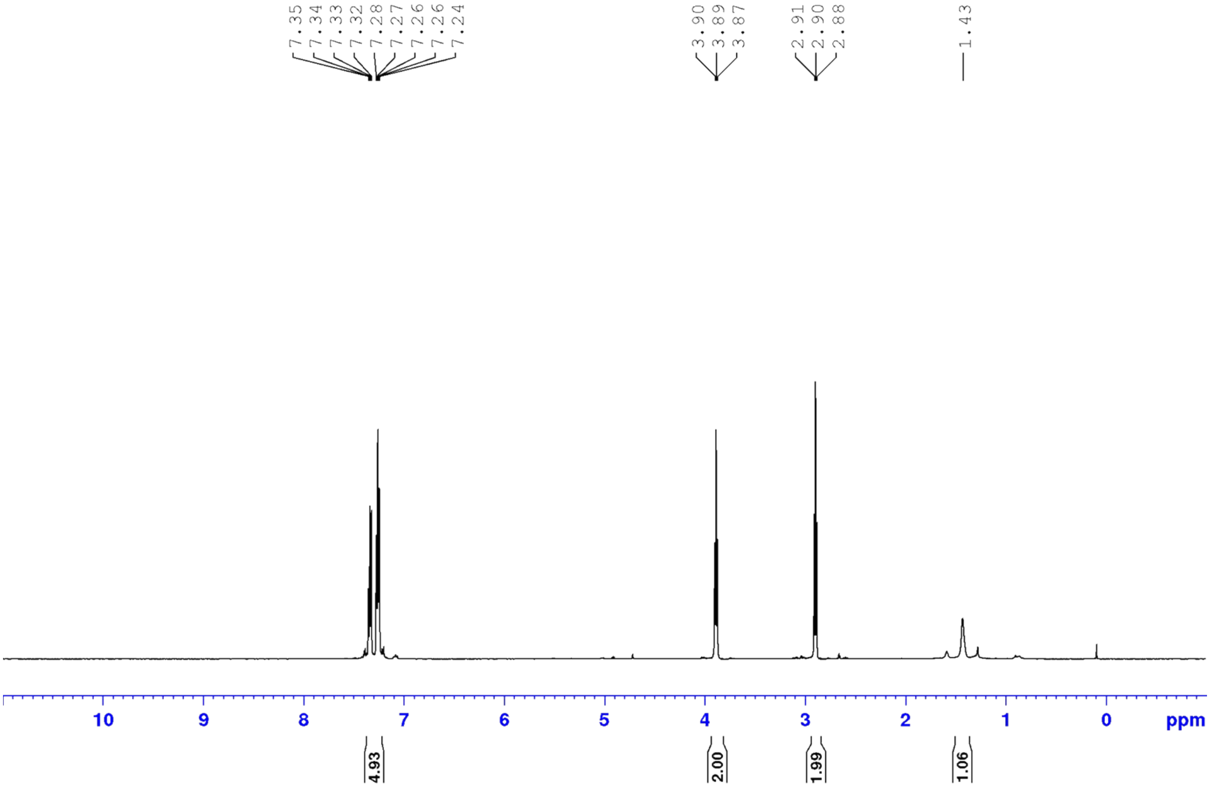

Figure S17: ^{1}H NMR of extracted 2-phenylethyl alcohol [$CDCl_3$, 500 MHz]

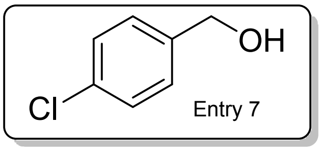

Entry 7: 100 mg (0.027 mmol) of [Fe_4L_6] cage, 42 mg (0.3 mmol) of 4-chlorobenzaldehyde and 19 mg (0.3 mmol) of $NaCNBH_3$ were taken in 5 mL distilled water under a nitrogen atmosphere and stirred for 6 hours at 50 °C. Yield: 52% (22 mg, 0.15 mmol). ^{1}H NMR (500 MHz, $CDCl_3$) δ = 7.33-7.28 (m, 4H), 4.66 (s, 2H), 1.85 (br s, 1H). ^{13}C NMR (126 MHz, $CDCl_3$) δ = 139.40, 133.50, 128.82, 128.41, 64.68. Mass spectroscopy (ESI): mass calculated for C_7H_7ClNaO ([M+Na]$^+$): 165.0083; Mass found: 165.0068.

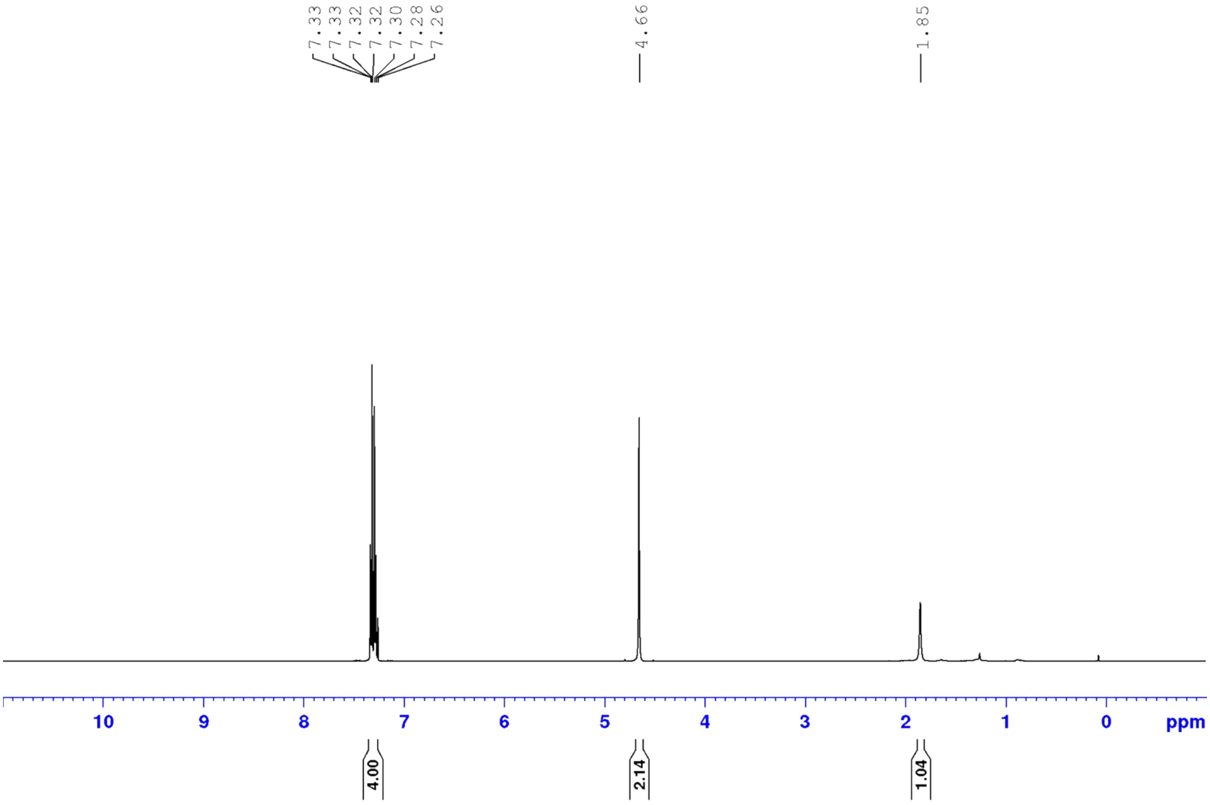

Figure S18: ^{1}H NMR of extracted 4-chlorobenzyl alcohol [$CDCl_3$, 500 MHz]

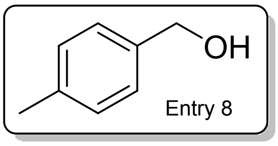

Entry 8: 100 mg (0.027 mmol) of [Fe_4L_6] cage, 36 mg (0.3 mmol) of 4-methylbenzaldehyde and 19 mg (0.3 mmol) of $NaCNBH_3$ were taken in 5 mL distilled water under a nitrogen atmosphere and stirred for 6 hours at 50 °C. Yield: 39% (14 mg, 0.12 mmol). ^{1}H NMR (500 MHz, $CDCl_3$) δ = 7.27 (d, *J* = 7.8 Hz, 2H), 7.18 (d, *J* = 7.8 Hz, 2H), 4.64 (s, 2H), 2.36 (s, 3H), 1.69 (br s, 1H). ^{13}C NMR (126 MHz, $CDCl_3$) δ = 138.09, 137.57, 129.41, 127.29, 65.44, 21.31. Mass spectroscopy (ESI): mass calculated for $C_8H_{10}NaO$ ($[M+Na]^+$): 145.0629; Mass found: 145.0609.

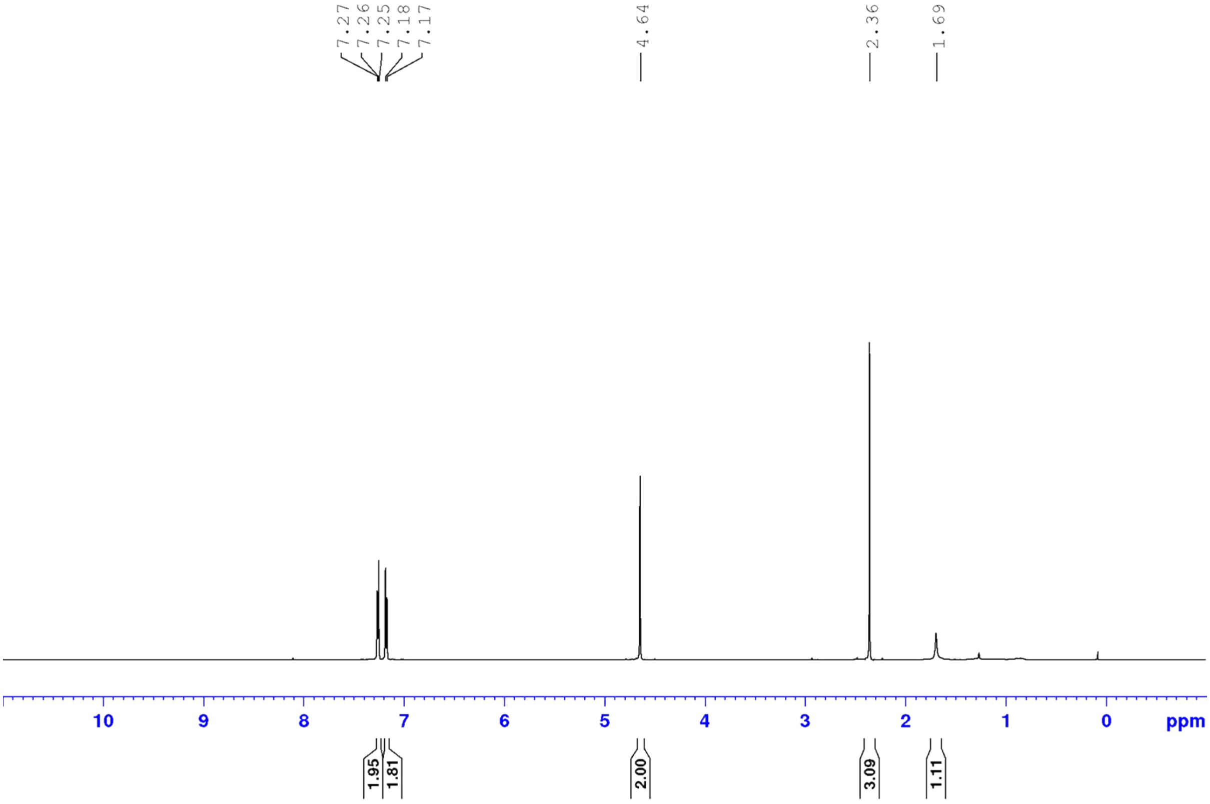

Figure S19: ^{1}H NMR of extracted 4-methylbenzyl alcohol [$CDCl_3$, 500 MHz]

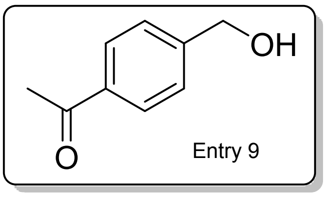

Entry 9: 100 mg (0.027 mmol) of $[Fe_4L_6]$ cage, 45 mg (0.3 mmol) of 4-acetylbenzaldehyde and 19 mg (0.3 mmol) of $NaCNBH_3$ were taken in 5 mL distilled water under a nitrogen atmosphere and stirred for 6 hours at 50 °C. Yield: 60% (27 mg, 0.18 mmol). ^1H NMR (400 MHz, $CDCl_3$) δ = 7.97 (d, *J* = 8.3 Hz, 2H), 7.45 (d, *J* = 8.3 Hz, 2H), 4.79 (s, 2H), 2.61 (s, 3H), 1.56 (br s, 1H). ^{13}C NMR (101 MHz, $CDCl_3$) δ = 197.8, 146.1, 136.5, 128.6, 126.6, 64.7, 26.6. Mass spectroscopy (ESI): mass calculated for $C_9H_{10}NaO_2$ ($[M+Na]^+$): 173.0579; Mass found: 173.0571.

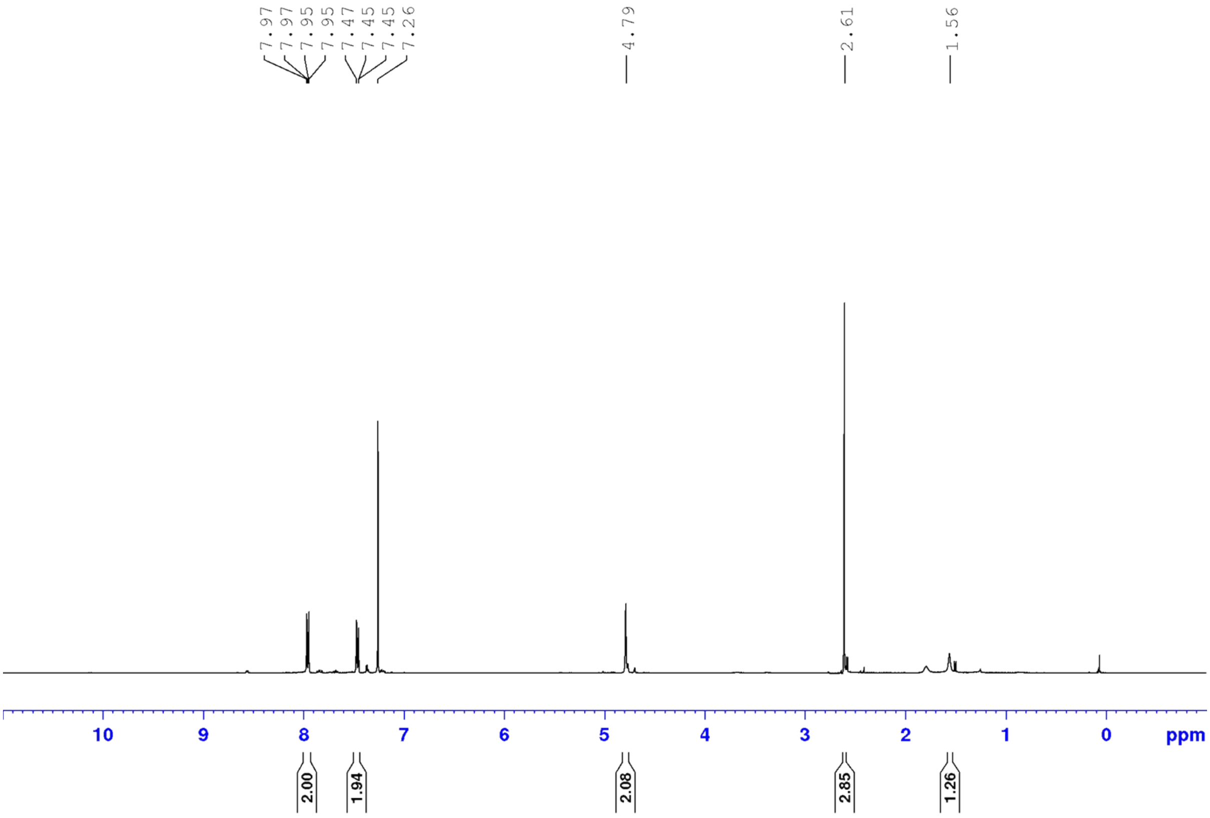

Figure S20: ^1H NMR of extracted 1-[4-(hydroxymethyl)phenyl]ethanone [$CDCl_3$, 400 MHz]

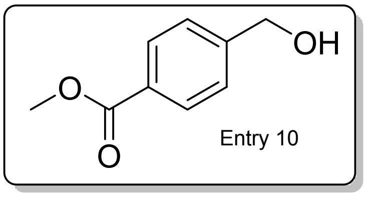

Entry 10: 100 mg (0.027 mmol) of [Fe_4L_6] cage, 49 mg (0.3 mmol) of methyl 4-formylbenzoate and 19 mg (0.3 mmol) of $NaCNBH_3$ were taken in 5 mL distilled water under a nitrogen atmosphere and stirred for 6 hours at 50 °C. Yield: 63% (31 mg, 0.19 mmol). ^{1}H NMR (400 MHz, $CDCl_3$) δ = 8.05 (d, *J* = 8.4 Hz, 2H), 7.43 (d, *J* = 8.4 Hz, 2H), 4.78 (s, 2H), 3.92 (s, 3H), 1.55 (br s, 1H). ^{13}C NMR (101 MHz, $CDCl_3$) δ = 166.9, 145.9, 129.9, 129.4, 126.5, 64.8, 52.1. Mass spectroscopy (ESI): mass calculated for $C_9H_{10}NaO_3$ ([M+Na]$^+$): 189.0528; Mass found: 189.0518.

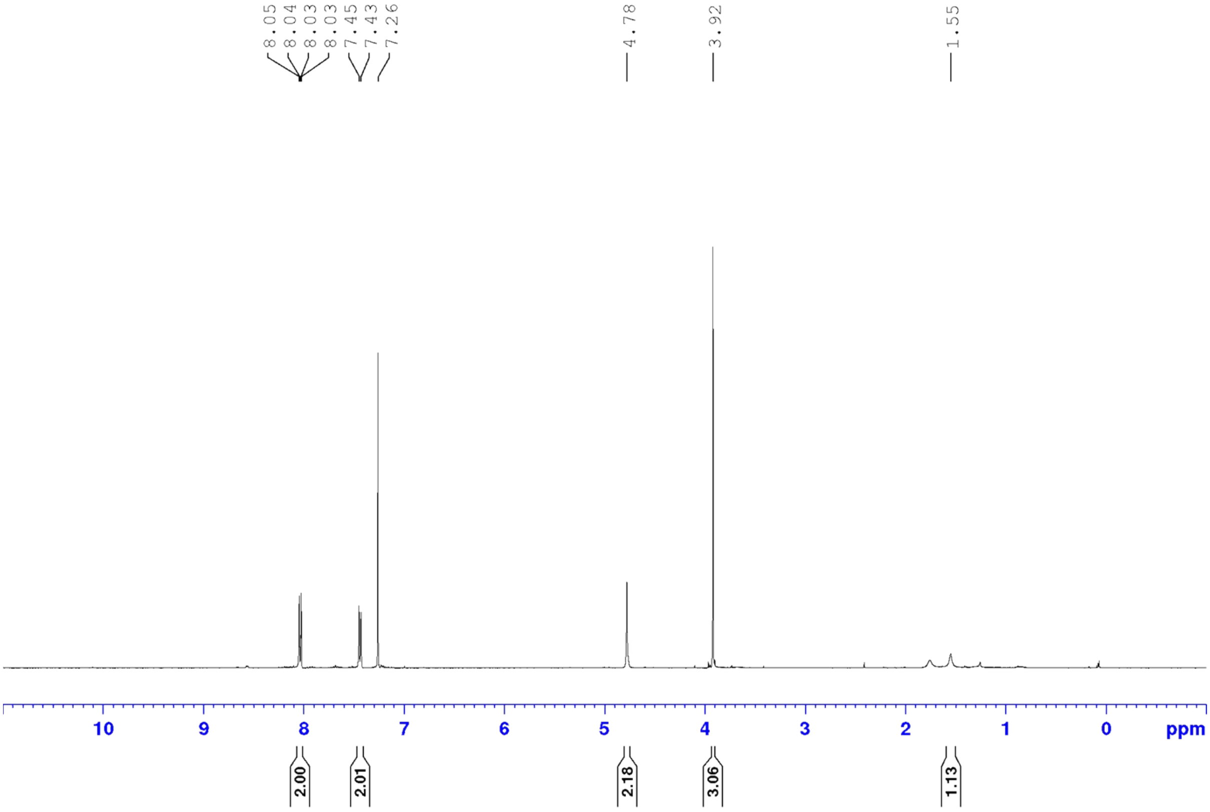

Figure S21: ^{1}H NMR of extracted methyl 4-(hydroxymethyl)benzoate [$CDCl_3$, 400 MHz]

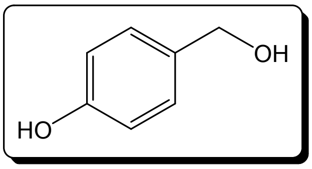

Entry 11: 100 mg (0.027 mmol) of $[Fe_4L_6]$ cage, 37 mg (0.3 mmol) of 4-hydroxybenzaldehyde and 19 mg (0.3 mmol) of $NaCNBH_3$ were taken in 5 mL distilled water under a nitrogen atmosphere and stirred for 6 hours at 50 °C. Yield: 67% (25 mg, 0.20 mmol). ^1H NMR (500 MHz, DMSO-d_6) δ = δ 9.22 (s, 1H), 7.09 (d, J = 8.4 Hz, 2H), 6.69 (d, J = 8.4 Hz, 2H), 4.93 (t, J = 5.7 Hz, 1H), 4.35 (d, J = 5.7 Hz, 2H). ^{13}C NMR (126 MHz, DMSO-d_6) δ = 156.1, 132.7, 128.0, 114.7, 62.7. Mass spectroscopy (ESI): mass calculated for $C_7H_8NaO_2$ ($[M+Na]^+$): 147.0422; Mass found: 147.0409.

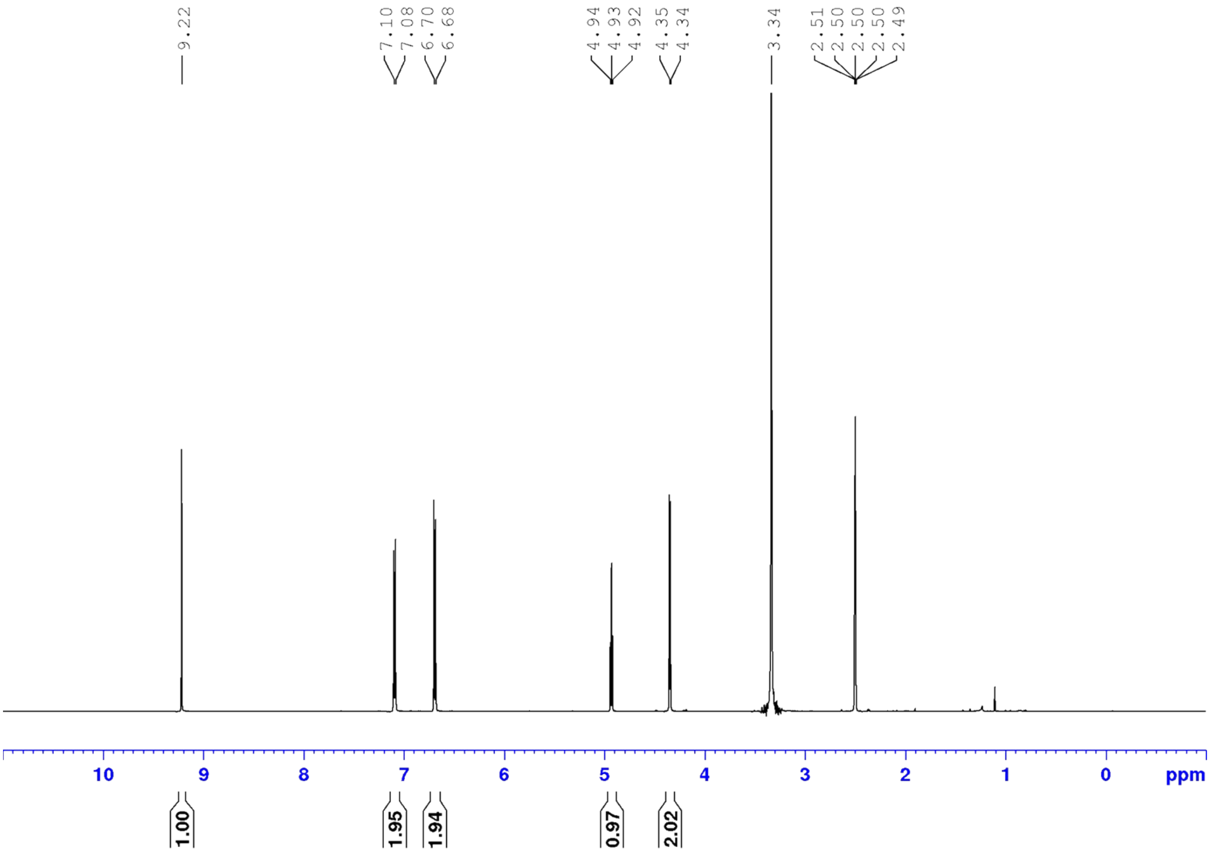

Figure S22: ^1H NMR of extracted 4-hydroxybenzyl alcohol [DMSO-d_6, 500 MHz].

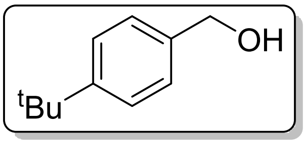

Entry 12: 100 mg (0.027 mmol) of $[Fe_4L_6]$ cage, 49 mg (0.3 mmol) of 4-tertbutylbenzaldehyde and 19 mg (0.3 mmol) of $NaCNBH_3$ were taken in 5 mL distilled water under a nitrogen atmosphere and stirred for 6 hours at 50 °C. Yield: 27% (13.3 mg, 0.08 mmol). ^{1}H NMR (400 MHz, $CDCl_3$) δ = 7.41 (d, J = 8.4 Hz, 2H), 7.32 (d, J = 8.4 Hz, 2H), 4.68 (d, J = 5.7 Hz, 2H), 1.61 (t, J = 5.7 Hz, 1H), 1.33 (s, 9H). ^{13}C NMR (101 MHz, $CDCl_3$) δ = 150.8, 137.9, 126.9, 125.5, 65.2, 34.6, 31.3. Mass spectroscopy (ESI): mass calculated for $C_{11}H_{16}NaO_3$ ($[M+Na]^+$): 187.1099; Mass found: 187.1098.

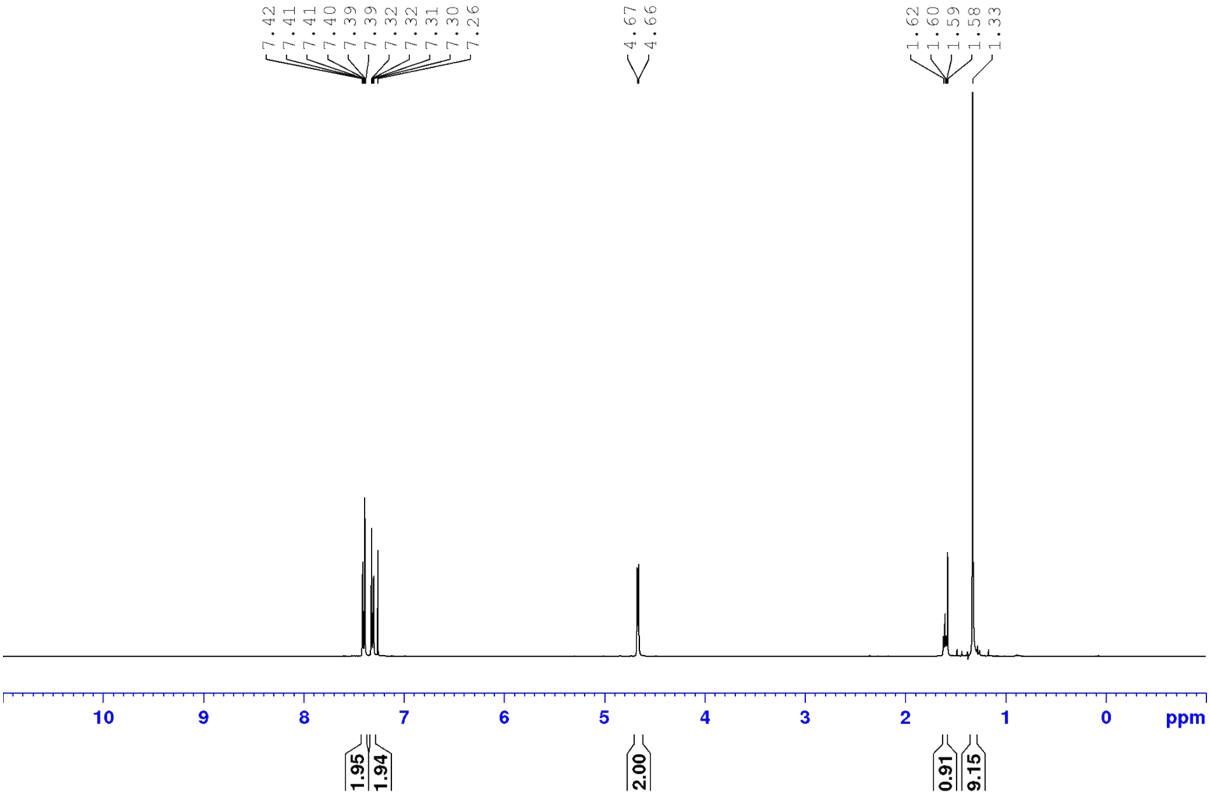

Figure S23: ^{1}H NMR of extracted methyl 4-tertbutylbenzyl alcohol [$CDCl_3$, 400 MHz]

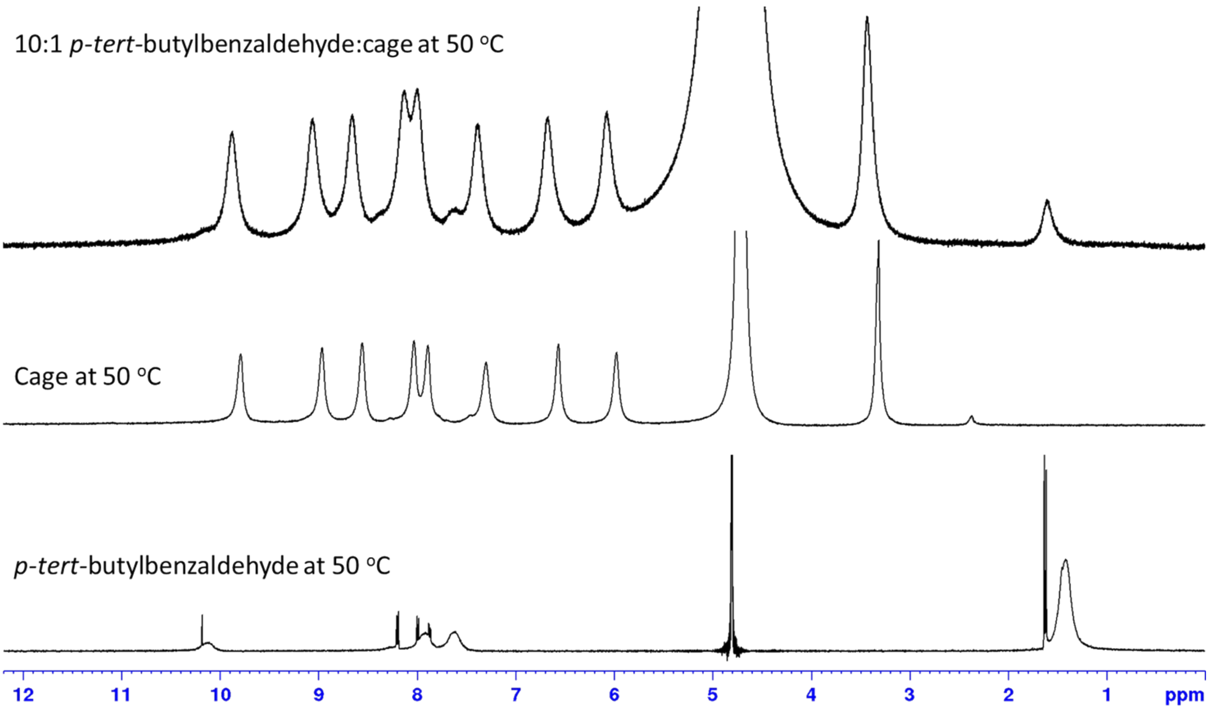

Figure S24: Stacked ^{1}H NMR spectra (500 MHz) in D_2O at 323 K of *p-tert*-butylbenzaldehyde on its own (bottom), $Fe^{II}_4L_6$ cage on its own (middle) and a 1:10 mixture of cage and *p-tert*-butylbenzaldehyde (top). Upon addition of the *p-tert*-butylbenzaldehyde to the cage, both sets of peaks broaden considerably, consistent with an intermediate rate of exchange on the NMR timescale.

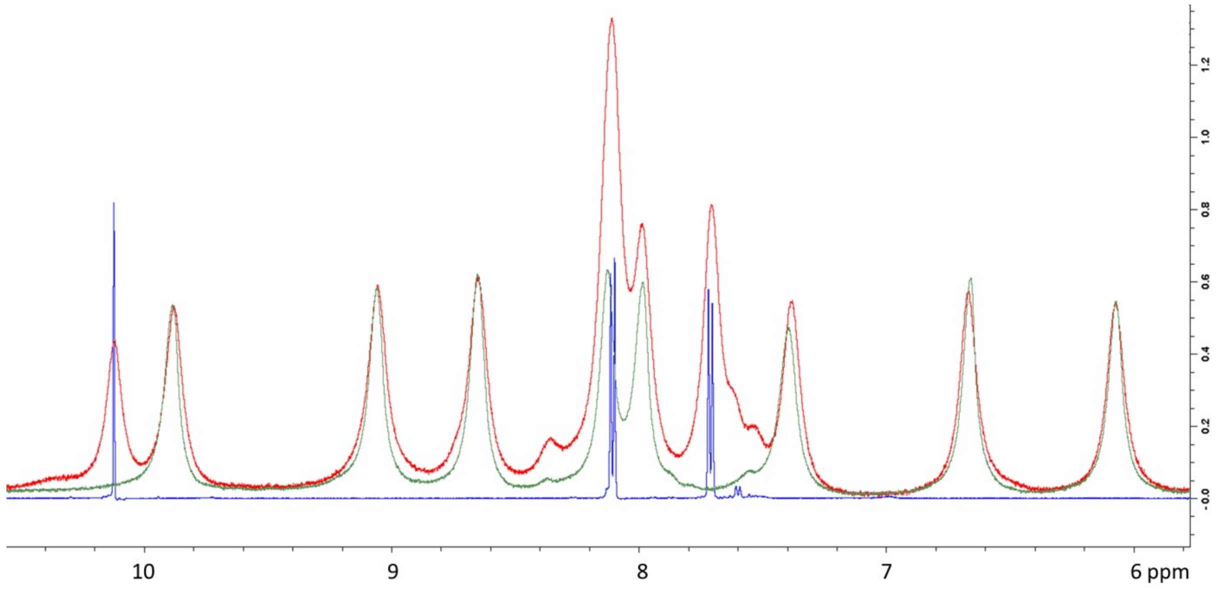

Figure S25: Overlaid ^{1}H NMR spectra (500 MHz) in D_2O at 323 K of *p*-tolualdehyde on its own (blue), $Fe^{II}_4L_6$ cage on its own (green) and a 1:10 mixture of cage and *p*-tolualdehyde (red). Upon addition of the *p*-tolualdehyde to the cage, both sets of peaks broaden considerably, consistent with an intermediate rate of exchange on the NMR timescale.

References

S1. P. Mal, D. Schultz, K. Beyeh, K. Rissanen, J. R. Nitschke, *Angew. Chem. Int. Ed.* **2008**, *47*, 8297.

S2. F. Neese, *WIREs Comput. Molec. Sci.* **2012**, *2*, 73.

S3. M. Thompson, 2005 *ArgusLab 4.01*. PlanariaSoftware LLC, Seattle, WA, USA. See http://www.ArgusLab.com.

S4. a) A. D. Becke, *Phys. Rev. A* **1988**, *38*, 3098; b) J. P. Perdew, *Phys. Rev. B* **1986**, *33*, 8822.

S5. A. Klamt, G. Schüürmann, *J. Chem. Soc., Perkin Trans. 2* **1993**, 799.

S6. a) A. Schäfer, H. Horn, R. Ahlrichs, *J. Chem. Phys.* **1992**, *97*, 2571; b) A. Schäfer, C. Huber, R. Ahlrichs, *J. Chem. Phys.* **1994**, *100*, 5829; c) F. Weigend, R. Ahlrichs, *Phys. Chem. Chem. Phys.* **2005**, *7*, 3297.

S7. F. Weigend, R. Ahlrichs, *Phys. Chem. Chem. Phys.* **2005**, *7*, 3297.

S8. a) S. Grimme, J. Antony, S. Ehrlich, H. Krieg, *J. Chem. Phys.* **2010**, *132*, 154104; b) S. Grimme, S. Ehrlich, L. Goerigk, *J. Comput. Chem.* **2011**, *32*, 1456.

S9. a) E. van Lenthe, J. G. Snijders, E. J. Baerends, *J. Chem. Phys.* **1996**, *105*, 6505; b) E. van Lenthe, A. van der Avoird, P. E. S. Wormer, *J. Chem. Phys.* **1998**, *108*, 4783; c) J. H. van Lenthe, S. Faas, J. G. Snijders, *Chem. Phys. Lett.* **2000**, *328*, 107.

S10. C. J. van Wüllen, *J. Chem. Phys.* **1998**, *109*, 392.

S11. F. Neese, *J. Comput. Chem.* **2003**, *24*, 1740.

S12. a) P. Pulay, *Chem. Phys. Lett.* **1980**, *73*, 393; b) P. Pulay, *J. Comput. Chem.* **1982**, *3*, 556.

S13. *Molekel*, Advanced Interactive 3D-Graphics for Molecular Sciences, Swiss National Supercomputing Center. https://ugovaretto.github.io/molekel/
